# Multimodal prehabilitation is an effective strategy to reduce postoperative complications and improve physical function and anxiety in patients with colorectal cancer undergoing elective surgery: a systematic review and network meta-analysis

**DOI:** 10.3389/fmed.2025.1619959

**Published:** 2025-10-01

**Authors:** Na Li, Xiaoli Liu, Yanping Wang, Ruimei Song, Xufeng Xie

**Affiliations:** ^1^Department of Nursing, Tongji University School of Medicine, Shanghai, China; ^2^Department of Nursing, Shanghai Prison General Hospital, Shanghai, China; ^3^Department of Hepatobiliary Surgery, Tenth People’s Hospital of Tongji University, Shanghai, China; ^4^Department of Rehabilitation Medicine, The Affiliated Hospital of Southwest Medical University, Shanghai, China

**Keywords:** colorectal cancer, prehabilitation, postoperative outcomes, multimodal intervention, network meta-analysis

## Abstract

**Background:**

Preoperative prehabilitation represents a viable approach to improve postoperative recovery and quality of life in colorectal cancer (CRC) patients, though debates persist regarding the efficacy of specific prehabilitation modalities.

**Objective:**

This study aims to compare and rank prehabilitation strategies for enhancing postoperative outcomes in CRC patients through a network meta-analysis, identifying the optimal preoperative prehabilitation method.

**Methods:**

We included randomized controlled trials (RCTs) assessing four prehabilitation strategies in CRC patients. Outcome measures focused on postoperative complications, hospital stay duration, 6-min walk test, and states of anxiety and depression. The effect sizes for dichotomous outcomes were measured by odds ratios (OR), and for continuous outcomes by mean differences (MD) or standardized mean differences (SMD), with 95% credible intervals (CrIs).

**Results:**

Our analysis included 27 RCTs involving 2,946 CRC patients. NMA results indicated that, compared to the control group (CON), only the multimodal (Mul) approach significantly reduced postoperative complications (OR: 0.47, 95%CrI: 0.26–0.85) and hospitalization time (MD: −1.17, 95CrI: −1.77 to −0.57). Moreover, Mul was the only strategy that improved pre-surgical 6-min walk test results (MD: 27.22, 95CrI: 12.71–41.73) and anxiety levels (SMD: −0.69, 95CrI: −1.34 to −0.04), with sustained improvements in the 6-min walk test observed up to 4 weeks post-surgery (MD: 19.22, 95CrI: 5.94–32.50).

**Conclusion:**

The Mul prehabilitation program is the effective strategy for improving surgical outcomes in CRC patients. This comprehensive approach not only aids in reducing postoperative complications and shortening hospital stays but also enhances physical and psychological readiness before surgery.

**Systematic review registration:**

https://www.crd.york.ac.uk/PROSPERO/view/CRD42024514661.

## 1 Introduction

In recent years, prehabilitation has garnered widespread attention as an innovative intervention strategy. Prehabilitation encompasses a comprehensive suite of interventions implemented before surgery, designed to enhance patients’ physical and psychological states, thereby aiming to mitigate postoperative complications, expedite the recovery process, and improve overall rehabilitation quality (1, 2). These interventions typically include physical exercise, nutritional support, psychological interventions, and social support mechanisms. Current research indicates that prehabilitation can effectively improve patients’ preoperative health status, reduce the risk of postoperative complications, shorten hospital stays, and contribute to an enhanced quality of life (3). This multidisciplinary approach not only prepares patients for the physiological demands of surgery but also addresses the psychological and social aspects of recovery, offering a holistic strategy to improve surgical outcomes and patient wellbeing.

In the domain of prehabilitation for Colorectal cancer (CRC) surgery, evidence suggests that preoperative interventions can enhance functional capacity and potentially reduce the incidence of postoperative complications and emergency visits. Studies have demonstrated that engaging patients in prehabilitation programs prior to surgery can lead to improved functional outcomes and a decrease in the likelihood of post-surgical complications (2). However, meta-analyses have yielded mixed results, with some finding that prehabilitation does not significantly impact postoperative complications, length of hospital stay, or functional abilities for CRC surgery patients (4). Furthermore, research on prehabilitation interventions for CRC surgery has predominantly focused on various individualized approaches, including physical training, nutritional interventions, psychological support, or a combination thereof (5). Each of these prehabilitation modalities offers distinct benefits: physical training can enhance patients’ physical fitness and immunity (6); nutritional interventions may improve nutritional status (7); and psychological support can alleviate preoperative anxiety and depression (8). Despite these advantages, there remains a considerable gap in the literature regarding whether prehabilitation can improve postoperative physical and psychological outcomes for CRC patients and which prehabilitation approach is most effective for enhancing recovery outcomes.

The systematic review and network meta-analysis to compare the effects of various prehabilitation interventions on postoperative complications, length of hospital stay, physical function, and mood among patients undergoing CRC surgery. By conducting a thorough analysis and synthesis of the existing literature, this research aims to identify the most effective prehabilitation strategy for enhancing postoperative outcomes.

## 2 Methods

This pre-registered systematic review with network meta-analysis (PROSPERO reference number # CRD42024514661) adhered to the reporting requirements outlined in the PRISMA checklist (9).

### 2.1 Search strategy

We performed a systematic search across PubMed, MEDLINE, Embase, Cochrane Central Register of Controlled Trials (CENTRAL), and Web of Science from their inception until February 3, 2025, without language restrictions. Detailed search strategies, encompassing search terms, dates, and methodology, are outlined in Supplementary File 1. Additionally, we reviewed the reference lists of relevant articles and reviews to identify further studies. Both title/abstract and full-text screening were independently carried out in duplicate by investigators. Any discrepancies were resolved through discussion or, if necessary, by the intervention of a third author for adjudication.

### 2.2 Study selection

The initial screening of all identified abstracts was per- formed independently by 2 reviewers. We included randomized controlled trials (RCTs) on the effects of prehabilitation on adult patients scheduled for elective surgical resection of primary CRC. The intervention included 4 types of prehabilitation: breath-only training (BT), exercise-only (EX, included aerobic and total body resistance exercise), multimodal prehabilitation programme (exercise, nutritional, and anxiety-reduction strategies) (Mul), nutrition-only prehabilitation (NU, Nutritional prehabilitation entails non-invasive dietary adjustments through supplements and/or counseling to optimize macronutrient intake for at least a week before surgery). The comparators included those receiving standard care, or an active control, which could involve a different prehabilitation type from the intervention group.

### 2.3 Outcomes

The primary outcome was 30 days postoperative complications, was recorded in number of people in the incident, and the length of hospital stay after colorectal surgery, was recorded in mean days, beginning the day of surgery until hospital discharge. In addition, secondary outcomes include changes in physical function and mood. In terms of functional ability, we evaluated the effects of preoperative prehabilitation on the 6-min walk test (6 WMT), and maintenance at 4 and 8 weeks after surgery compared with baseline. For mood, we assessed anxiety and depressive symptoms on pre-surgery, post-surgery 4 and 8 weeks.

### 2.4 Risk of bias

Two authors evaluated the risk of bias in the studies using the revised Cochrane risk of bias tool (RoB 2 tool) at the study level (10), and for the following domains: randomization process, deviations from intended interventions, missing outcome data, measurement of the outcome, and selection of the reported result. Any disagreements in assessment were resolved by consulting a third reviewer.

### 2.5 Data synthesis

#### 2.5.1 Pairwise meta-analysis

For outcomes that were influenced by only one type of prehabilitation intervention, we conducted a pairwise meta-analysis comparing this to standard care. Analyses were performed in R (V.4.3.2)^1^ using a random-effects model within the “metafor” package. We visually assessed the heterogeneity of treatment effects through forest plots, closely monitoring τ^2^ and the I^2^ statistic. Prediction intervals were also included in the forest plots to better illustrate heterogeneity.

#### 2.5.2 Assessment of the transitivity assumption

Transitivity is the key underlying assumption of network meta-analysis (11). To assess this assumption, we examined the distribution of possible effect modifiers across treatment comparisons. Potential effect modifiers included laparoscopic surgery proportion, sample size, mean age, percentage female, prehabilitation duration. We use the R ggplot2 package to draw boxplots between the above potential influencing factors and various types of prehabilitation. At the same time, we analyzed the differences in the above potential influencing factors between various prehabilitation programs through one-way ANOVA analysis (Supplementary File 2).

#### 2.5.3 Network meta-analysis

A network plot was generated utilizing Stata software (version 14, StataCorp LLC, TX, United States) to visually represent the network of comparisons across trials, ensuring the viability of the network meta-analyses. In the context of comparing the effects of various prehabilitation types, we conducted Bayesian network meta-analyses using the gemtc and rjags packages within the R statistical environment (V.4.3.2) (see text footnote 1). This approach involves calculating the posterior distribution of parameters based on the available data to update prior information, as Bayesian methods are more prevalent in such analyses compared to frequentist approaches. Markov chains were used to generate samples. Model convergence was assessed using the Brooks-Gelman-Rubin plots method. The effect sizes were calculated as odds ratio (OR) for dichotomous outcomes and mean differences (MD) for continuous outcomes. Due to anxiety and depressive symptoms were assessed by different scales, we chose the standard mean difference (SMD) to assess effect size. To evaluate the reliability of our estimates, we utilized 95% credible intervals (CrI). For data synthesis, a random-effects model was employed to combine the data, while the surface under the cumulative ranking (SUCRA) probabilities was utilized to rank the various treatments.

#### 2.5.4 Assessment of heterogeneity and inconsistency

Statistical heterogeneity between studies was examined using the τ^2^ test and I^2^ statistics. Statistical consistency was evaluated using the design-by-treatment test (12) and by differentiating indirect from direct evidence (SIDE test) (13) via the R “netmeta” package. We further conducted a comparison using adjusted funnel plots to assess potential publication bias under specific circumstances (14). Egger’s test was employed to indicate potential publication bias when *p* < 0.05.

#### 2.5.5 Sensitivity analysis

We only performed sensitivity analysis on the primary outcome. We hypothesized that the quality of included literature might contribute to heterogeneity and inconsistency. Thus, we assessed the sensitivity of our findings by repeating network meta-analysis after excluding high risk studies.

## 3 Results

**Figure 1:**
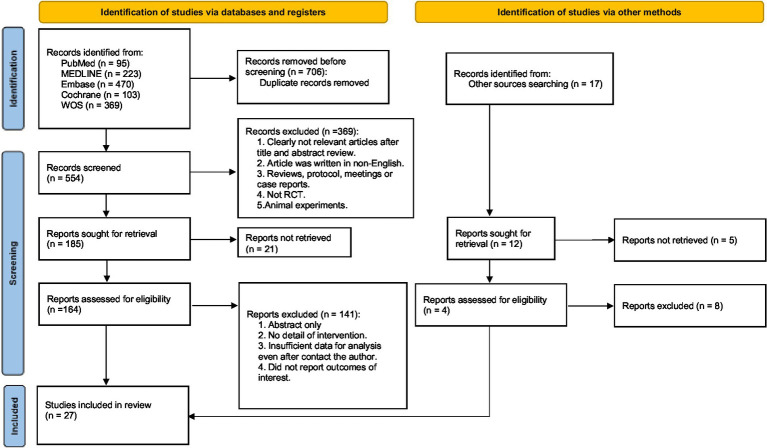
PRISMA flow diagram of the search process for studies.

Overall, 1,260 records were identified through the initial electronic searches. After removing duplicates, 554 records were screened for titles and abstracts and 164 full-text articles were screened for eligibility. In total, 27 studies involving 2,946 participants were included in the review (Figure 1).

### 3.1 Assessment of the transitivity assumption

Potential threats to the transitivity assumption and the source of heterogeneity from baseline characteristics (laparoscopic surgery proportion, sample size, mean age, percentage female, prehabilitation duration) of the included studies were resolved by one-way ANOVA (Supplementary File 2:Figures 2.1–2.5). No significant factors affecting the network meta-analysis results.

### 3.2 Characteristics of included studies

The characteristics of included studies were shown in (Supplementary File 3). A total of 670 participants across 11 studies underwent EX, 13 studies with 657 participants underwent Mul, 3 studies with 94 participants underwent NU, 1 study with 92 participants underwent BT, 26 studies with 1,433 participants underwent standard care. The sample size of the included studies ranged from 7 to 351, with a median of 40. The mean age ranged from 53.86 to 83.50, with a median of 67.6. The year of publication ranged from 2000 and 2023, with a median of 2020.

### 3.3 Risk of bias

Of the 27 trials, for overall bias, 14 studies were assessed as low risk of bias, 11 as some concerns and two as high. In the randomization process, 22 trials (81.5%) were low risk, five trials (18.5%) were some concerns; for deviations from intended interventions, 24 trials (88.9%) were at low risk, three trials (11.1%) were some concerns; in the missing outcome data, 22 trials (81.5%) were low risk, two trials (7.4%) were some concerns, and two trials (7.4%) were high risk; in the measurement of the outcome, 26 trials (96.3%) were low risk, one trial (3.7%) was some concerns; in the selection of the reported result, 25 trials (92.6%) were low risk, two trials (7.4%) were some concerns (Supplementary File 4).

### 3.4 Assessment of heterogeneity and inconsistency

For heterogeneity, most outcomes were high (Supplementary File 5:Table 5.1). It was worth noting that no significant inconsistencies were found in the evaluated outcomes according to the design-by-treatment interaction test (Supplementary File 5:Table 5.2). At the same time, the SIDE test of all outcomes also found no statistical difference (Supplementary File 5:Tables 5.3.1–3.9). Additionally, our comparison-adjusted funnel plot had good symmetry for all outcomes, and the results of Egger’s test (*p* > 0.05) showed that no small study effect was found (Supplementary File 8:Figures 8.1–8.11).

### 3.5 The results of meta-analysis

#### 3.5.1 Primary outcome

**Figure 2:**
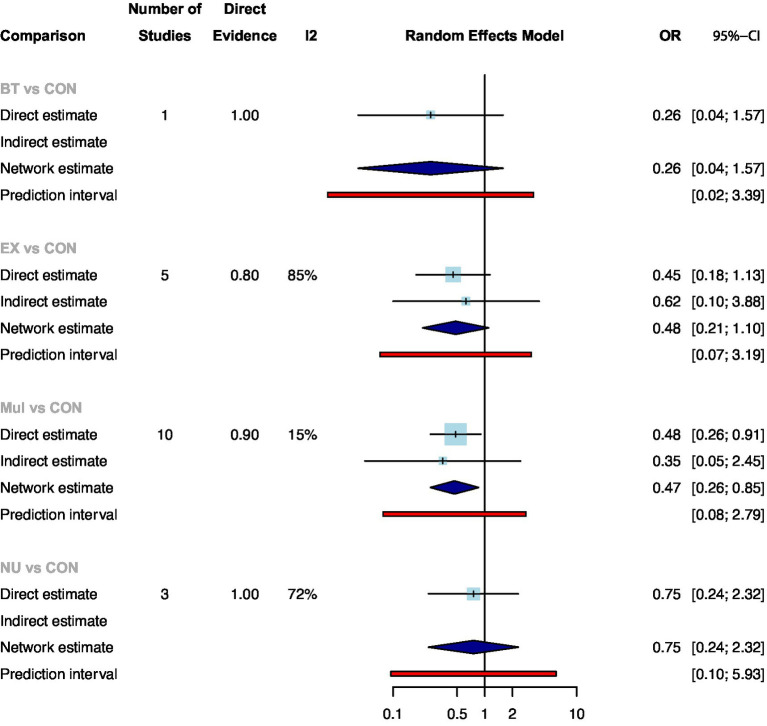
Summary of network meta-analysis results on postoperative complications for each type of prehabilitation compared with controls.

Twenty of the studies with 1816 participants assessed postoperative complications. Supplementary File 6:Figure 6.1 showed the direct comparison and sample size distribution between the prehabilitation types for postoperative complications. The results of network meta-analysis showed that only Mul significantly reduced the postoperative complications compared with standard care (Figure 2: OR: 0.47, 95%CrI: 0.26–0.85). Ranking according to the degree of postoperative complications, BT was the best and standard care was the worst (Supplementary File 7:Table 7.1). In addition, no statistical differences were found between the prehabilitation types.

**Figure 3:**
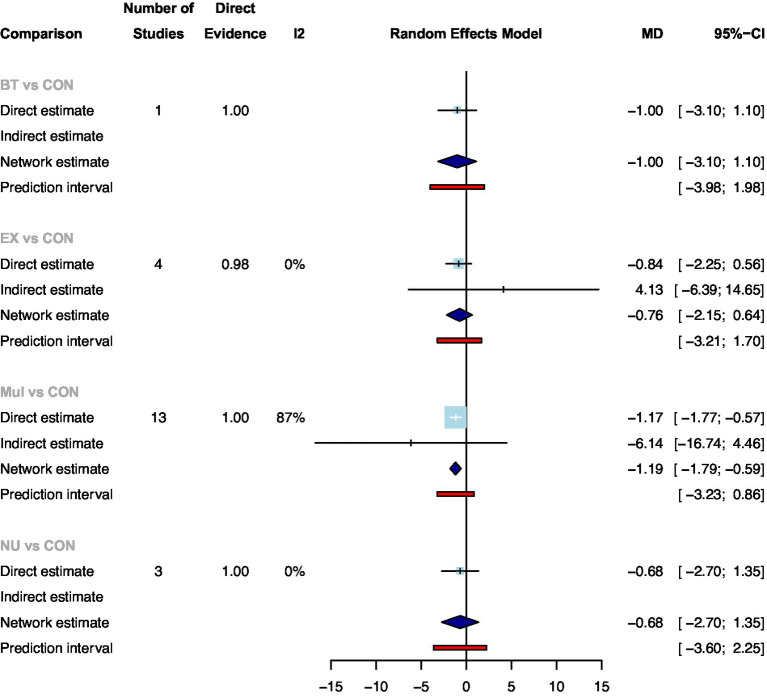
Summary of network meta-analysis results on the length of hospital stay for each type of prehabilitation compared with controls.

A total of 22 studies with 2050 participants showed available results for the length of hospital stay after colorectal surgery (Supplementary File 6:Figure 6.2). Compared with standard care, only Mul significantly reduced the length of hospital stay (Figure 3: MD: −1.17, 95CrI: −1.77 to −0.57). Ranking according to the degree of length of hospital stay, Mul was the best and standard care was the worst (Supplementary File 7:Table 7.2). In addition, no statistical differences were found between the prehabilitation types.

#### 3.5.2 Secondary outcomes

Results for secondary outcome measures were summarized in Table 1. Fifteen studies with 1,082 participants showed available results for the 6 WMT pre-surgery (Supplementary File 6:Figure 6.3). Compared with standard care, only Mul significantly improved the 6 WMT (Supplementary File 7:Table 7.3: MD: 27.22, 95CrI: 12.71–41.73). Ranking according to the degree of 6 WMT, Mul was the best and standard care was the worst. In addition, no statistical differences were found between the prehabilitation types. At the same time, the 6 WMT post-surgery 4 weeks (Supplementary File 6:Figure 6.4: 10 studies with 995 participants) was only Mul significantly maintained compared with standard care (Supplementary File 7:Table 7.4: MD: 19.22, 95CrI: 5.94–32.50). When 8 weeks after surgery (Supplementary File 6:Figure 6.5: 6 studies with 585 participants), no prehabilitation was found to significantly maintain the effect (Supplementary File 7: Table 7.5).

**Table 1:** Secondary outcomes of prehabilitation to postoperative outcomes in colorectal cancer patients.

Outcome		Studies in pairwise treatment comparison (participant*)	Network meta-analysis		
			Network estimate on MD or SMD (95% CrI)	Direct estimate on MD or SMD (95% CrI)	SUCRA
6 WMT*	Pre-surgery				
	Mul vs. CON	7 (573)	**27.22 (12.71; 41.73)**	15.64 (−17.12; 48.40)	0.85
	NU vs. CON	1 (43)	19.60 (−23.52; 62.72)	19.60 (−23.52; 62.72)	0.61
	EX vs. CON	6 (323)	11.15 (−11.79; 34.08)	15.64 (−17.12; 48.40)	0.43
	Post-surgery 4 weeks				
	EX vs. CON	1 (21)	65.00 (−85.88; 215.88)	65.00 (−85.88; 215.88)	0.74
	Mul vs. CON	5 (447)	**19.22 (5.94; 32.50)**	**19.22 (5.94; 32.50)**	0.60
	NU vs. CON	1 (43)	17.60 (−56.52; 91.72)	17.60 (−56.52; 91.72)	0.48
	Post-surgery 8 weeks				
	Mul vs. CON	5 (453)	24.41 (−7.26; 56.08)	24.41 (−7.26; 56.08)	0.86
	EX vs. CON	1 (112)	2.11 (−61.19; 65.41)	NA	0.37
Anxiety symptoms	Pre-surgery				
	Mul vs. CON	5 (462)	**−0.69 (−1.34; −0.04)**	**−0.74 (−1.41; −0.06)**	0.87
	EX vs. CON	2 (222)	−0.26 (−1.44; 0.93)	−0.00 (−1.61; 1.61)	0.45
	Post-surgery 4 weeks				
	Mul vs. CON	3 (297)	NA	−0.64 (−3.09; 1.82)	NA
	Post-surgery 8 weeks				
	Mul vs. CON	3 (285)	−0.08 (−0.32; 0.15)	−0.08 (−0.32; 0.15)	0.70
	EX vs. CON	1 (112)	−0.01 (−0.45; 0.43)	NA	0.44
Depressive symptoms	Pre-surgery				
	Mul vs. CON	5 (462)	−0.37 (−0.84; 0.11)	−0.42 (−0.93; 0.08)	0.79
	EX vs. CON	2 (222)	−0.23 (−0.95; 0.50)	−0.06 (−0.93; 0.82)	0.55
	Post-surgery 4 weeks				
	Mul vs. CON	3 (297)	NA	−0.53 (−3.62; 2.55)	NA
	Post-surgery 8 weeks				
	EX vs. CON	3 (259)	−0.09 (−0.60; 0.43)	NA	0.66
	Mul vs. CON	5 (462)	−0.02 (−0.29; 0.25)	−0.02 (−0.29; 0.25)	0.37

The asterisk represents that the effect value is MD. Cells shown in bold indicate significant results. SUCRA the surface under the cumulative ranking probabilities. BT breath training, CON standard care, EX exercise, Mul multimodal prehabilitation programme (exercise, nutritional, and anxiety-reduction strategies), NU nutritional intervention, NA not available.

Eight studies with 764 participants showed available results for the anxiety symptoms pre-surgery (Supplementary File 6:Figure 6.6). Compared with standard care, only Mul significantly relieved the anxiety symptoms (Supplementary File 7:Table 7.6: SMD: −0.69, 95CrI: −1.34 to −0.04). Ranking according to the degree of anxiety symptoms, Mul was the best and standard care was the worst. In addition, no statistical differences were found between the prehabilitation types. However, the effect was not maintained at 4 weeks after surgery (Supplementary File 9: Figure 9.1: analysis by pairwise meta-analysis as only one type of prehabilitation was compared with standard care), and 8 weeks after surgery (Supplementary File 6:Figure 6.7 and Supplementary File 7:Table 7.7).

Nine studies with 801 participants showed available results for the depressive symptoms pre-surgery (Supplementary File 6:Figure 6.8). Prehabilitation was not found to reduce symptoms of depression in CRC patients compared with standard care (Supplementary File 7:Table 7.8). No statistical difference was found 4 weeks (Supplementary File 9:Figure 9.2: analysis by pairwise meta-analysis as only one type of prehabilitation was compared with standard care) and 8 weeks after surgery (Supplementary File 6:Figure 6.9 and Supplementary File 7:Table 7.9).

### 3.6 Sensitivity analysis

The sensitivity of our findings for primary outcomes was assessed by repeating analyses after the exclusion of studies with high risk (two studies). The findings essentially remained the same in all sensitivity analyses (Supplementary File 10:Figures 10.1, 10.2), indicating that the inclusion of these studies did not have a major influence on results.

## 4 Discussion

Our study’s primary findings underscore the singular effectiveness of the Mul prehabilitation approach in significantly reducing postoperative complications and hospital stay durations among CRC patients, compared to standard care. This is in concordance with existing literature that highlights the advantages of a multimodal prehabilitation strategy in enhancing surgical outcomes. The research conducted by Molenaar et al. (15) have demonstrated similar reductions in postoperative complications, attributing these improvements to the comprehensive nature of Mul interventions that incorporate exercise, nutritional, and psychological support in a synergistic manner. The exercise component is recognized for boosting physical strength and immunity, thus reducing the risk of complications related to surgical immobility and infection (16). Nutritional interventions aim to ensure patients are in an optimal nutritional state to withstand the stresses of surgery and facilitate healing, thereby shortening recovery times and decreasing the likelihood of complications that could extend hospital stays (17). Psychological support, by mitigating preoperative anxiety and stress, potentially lowers the incidence of stress-related postoperative complications, such as delayed wound healing and impaired immune response (18). Furthermore, the progression from reduced postoperative complications to shortened hospital stays is logical and intuitive. A reduction in complications naturally lessens the need for prolonged medical interventions, directly resulting in shorter hospitalization periods (19). Hence, the observed significant reductions in postoperative complications and hospital stay durations can be attributed to the Mul approach’s integrated and synergistic method of combining physical, nutritional, and psychological support. This holistic strategy comprehensively addresses the complex needs of surgical patients, offering a more effective preparation for surgery and recovery than standard care alone.

The comprehensive benefits of the Mul prehabilitation strategy extend far beyond the medical outcomes for CRC patients, significantly enhancing both their functional and emotional states. Our research substantiates that Mul prehabilitation not only significantly improves CRC patients’ performance in the 6-min walk test but also effectively reduces their levels of postoperative anxiety. This evidence highlight the critical role of Mul’s multidimensional approach—encompassing physical exercise, nutritional advice, and psychological support—in achieving these improvements. In contrast, literature demonstrating limited or no impact of prehabilitation on the 6-min walk test and emotional states often describes interventions lacking this multimodal scope (20). This distinction highlights Mul’s unique advantage; whereas single-modality interventions might address specific care aspects, they do not adequately meet the comprehensive recovery needs of CRC patients (21). By integrating a range of supportive measures, Mul prehabilitation ensures a holistic recovery, promoting physical and emotional resilience in the preoperative phase. Additionally, the absence of significant improvements in depressive symptoms across various prehabilitation modalities, including Mul, suggests a preoperative prevalence of anxiety over depression (22), potentially explaining the observed ineffectiveness in mitigating depressive symptoms. This nuance underscores the necessity for targeted psychological interventions tailored to the specific emotional challenges encountered by patients before surgery, emphasizing the intricate nature of emotional well-being in surgical recovery and the essential need for customized prehabilitation strategies.

The maintenance of postoperative functional outcomes is critical for CRC patients, significantly impacting their rehabilitation trajectory and overall quality of life (23). Our study distinctly highlights that only the Mul prehabilitation strategy was effective in sustaining the improvements in the 6-min walk test at 4 weeks post-surgery, a benefit that did not extend to the 8-week mark. This observation underscores the 4-week postoperative period as a pivotal juncture in the recovery process, suggesting a nuanced interaction between the nature of colorectal surgery and the typical recovery timeline that may influence the observed duration of Mul’s benefits. Colorectal surgery, depending on its extent and complexity, generally entails a recovery period where significant improvements are observed within the first few weeks post-operation (24). This initial recovery phase is characterized by physical recuperation and gradual resumption of daily activities, where interventions like Mul can provide a marked advantage by enhancing physical capacity and resilience (25). However, as patients transition from the acute recovery phase toward more stable, long-term rehabilitation, the incremental benefits of pre-surgical conditioning, including those provided by Mul, may become less discernible. This transition is largely due to the body’s natural healing process and the regaining of pre-surgery physical function, which by the 8-week follow-up, results in a homogenization of functional abilities among patients, irrespective of their engagement in Mul prehabilitation. By the 8-week mark, patients typically approach a plateau in their recovery, where further improvements in physical function align more closely with the natural progression toward baseline health status rather than the direct influence of preoperative interventions (26). This plateau effect may explain why the significant disparities in physical function and recovery observed at 4 weeks post-surgery do not persist into later stages of recovery. Consequently, while Mul prehabilitation exhibits a clear advantage in the immediate postoperative period, its impact on sustained functional outcomes appears to diminish as patients collectively advance toward standardized recovery milestones, underscoring the temporal limits of prehabilitation interventions in the context of colorectal surgery recovery.

Additionally, the reason why any form of prehabilitation does not have a lasting effect on anxiety symptoms at 4 and 8 weeks post-surgery in CRC patients may lie in the psychological adjustments that occur postoperatively. Once the surgery is completed, the direct sources of pre-surgical anxiety, such as anticipation of the surgery and fears regarding complications, are likely either resolved or significantly alleviated (27). As patients move beyond the initial postoperative phase, their focus shifts toward recovery and adapting to life after surgery, potentially leading to a universal reduction in anxiety levels (28). This transition in focus, combined with the resolution of immediate surgical concerns, can explain why the initial improvements in anxiety symptoms facilitated by Mul prehabilitation do not exhibit long-term persistence when evaluated in the later postoperative period. This phenomenon underscores the complex nature of post-surgical psychological recovery, indicating that while preoperative interventions like Mul can provide temporary relief from anxiety, the evolving nature of patient concerns and psychological states post-surgery may diminish the lasting impact of such interventions on anxiety levels.

The strength of this research lies in its methodical and comprehensive evaluation of preoperative prehabilitation’s impact on CRC patients’ postoperative outcomes, utilizing a network meta-analysis to synthesize data from 27 randomized controlled trials involving 2,946 patients. By demonstrating significant benefits in reducing postoperative complications, shortening hospital stays, and improving both physical function and anxiety symptoms, this study highlights the potential of Mul prehabilitation as a critical strategy for optimizing surgical outcomes in CRC patients. However, the study is not without limitations. The heterogeneity introduced by the variability in the design and implementation of Mul interventions across trials may impact outcome comparability and the generalizability of results. Although statistical analyses were conducted to address this issue, differences in intervention specifics could limit the applicability of our findings. Furthermore, the follow-up period restricted to 8 weeks post-surgery constrains our capacity to assess the enduring effects of Mul prehabilitation, pointing to the transient nature of observed improvements in physical function and anxiety symptoms. Moreover, most included studies adopted different evaluation systems for postoperative complications, which limits further interpretation of the study results. Future studies should standardize complication grading and reporting as much as possible. Additionally, focusing exclusively on CRC patients restricts the direct applicability of our findings to this specific group, necessitating cautious extrapolation of results to other surgical cohorts. Future investigations should aim to assess the effectiveness of prehabilitation across a wider array of surgical populations and conditions, thereby expanding its applicability and utility in clinical practice.

## 5 Conclusion

This network meta-analysis of 27 randomized trials (2,946 colorectal cancer patients) reveals that multimodal prehabilitation—integrating exercise, nutrition, and psychological interventions—significantly reduces postoperative complications, shortens hospitalization, and enhances preoperative physical function and mental health, with benefits persisting post-surgery. These results underscore the necessity of adopting comprehensive prehabilitation protocols to optimize surgical recovery, minimize healthcare burdens, and improve outcomes in CRC patients.

## Data Availability

The raw data supporting the conclusions of this article will be made available by the authors, without undue reservation.

## Footnotes

^1^ www.r-project.org
