# Supplementary material for: Multimodal prehabilitation is an effective strategy to reduce postoperative complications and improve physical function and anxiety in patients with colorectal cancer undergoing elective surgery: a systematic review and network meta-analysis
Source: Front Med (Lausanne). 2025 Oct 1;12:1619959. doi: 10.3389/fmed.2025.1619959 (PMC12521261; doi:10.3389/fmed.2025.1619959)
Supplement: Supplementary file 1 [file Table_1.DOCX]

**Supplementary**

Table of Contents:

[Supplementary 1: Search Strategy 2](#_Toc194407223)

[Database: PubMed <inception to February 3 2024> 2](#_Toc194407224)

[Database: Ovid MEDLINE(R) <1946 to February 3 2025> 5](#_Toc194407225)

[Database: Embase <1974 to February 3 2025> 6](#_Toc194407226)

[Cochrane 7](#_Toc194407227)

[Database: Web of Science <1965 to February 3 2025> 8](#_Toc194407228)

[Supplementary 2: Assessment of the transitivity 9](#_Toc194407229)

[2.1 laparoscopic surgery proportion 9](#_Toc194407230)

[2.2 Mean age 10](#_Toc194407231)

[2.3 Percentage female 11](#_Toc194407232)

[2.4 Sample size 12](#_Toc194407233)

[2.5 Sample size 13](#_Toc194407234)

[Supplementary 3: Characteristics of studies and subjects included in the review 14](#_Toc194407235)

[Table 3.1: Characteristics of subjects included in the review 14](#_Toc194407236)

[Table 3.2 Characteristics of prehabilitation preogramme included in the review 16](#_Toc194407237)

[Table 3.3 Characteristics of outcomes included in the review 22](#_Toc194407238)

[Supplementary 4: Risk of Bias 28](#_Toc194407239)

[Table 4.1 The risk of bias assessment for the individual included studies 28](#_Toc194407240)

[Supplementary 5: Evaluation of heterogeneity and inconsistency 30](#_Toc194407241)

[Supplementary 6: Network plot 34](#_Toc194407242)

[Supplementary 7: League table 43](#_Toc194407243)

[Supplementary 8: Publication bias 52](#_Toc194407244)

[Supplementary 9: Forest plot of meta-analysis for pairwise comparisons 63](#_Toc194407245)

[Supplementary 10: Sensitivity analysis 64](#_Toc194407246)

# Supplementary 1: Search Strategy

## Database: PubMed <inception to February 3 2024>

***Search Strategy:***

| #15 | Search: ((((surg*) OR (resection)) OR (operat*)) AND (((surg*) OR (resection)) OR (operat*)) AND ((((((((((((abdo*) OR (bowel)) OR (Intestin*)) OR (colo*)) OR (duodenum)) OR (jejunum)) OR (rectum)) OR (proct*)) OR (sigmoid)) OR (anus)) OR (anal)) OR (nephr*) OR ((((laparotomy[MeSH Terms]) OR (laparoscopy[MeSH Terms])) OR (abdominal surgery)) OR (laparoscopic surgery)) AND (((((prehabilitation) OR (prehab*)) OR (pre-hab*)) OR ((((preop*) OR (pre-op*)) OR (pre-surg*)) OR (presurg*))) AND (((((((exercise[MeSH Terms]) OR ("physical activity")) OR (fitness)) OR (training)) OR (jogging)) AND (((((nutrition[MeSH Terms]) OR (diet*)) OR (nutri*)) OR (intake)) OR (supple*))) AND (((((((drug therapy) OR (pharmacotherapy)) OR (medication)) OR (drug)) OR (pharmac*)) OR (medicinal)) OR (medicine))))) AND (((((((recovery of function[MeSH Terms]) OR (postoperative complications[MeSH Terms])) OR (length of stay[MeSH Terms])) OR (quality of life[MeSH Terms])) OR (postoperative recovery)) OR (complication rate)) OR (hospital stay))) AND (("Randomized Controlled Trial"[Publication Type] OR "randomized"[Title/Abstract] OR "randomised"[Title/Abstract] OR "randomization"[Title/Abstract] OR "randomisation"[Title/Abstract] OR "Clinical Trials, Phase III as Topic"[MeSH] OR "Clinical Trials, Phase IV as Topic"[MeSH] OR randomly[Title/Abstract] OR trial*[Title] OR "Randomized Controlled Trials as Topic"[MeSH] OR randomized controlled trial*[Text Word] OR randomised controlled trial*[Text Word] OR randomized clinical trial*[Text Word] OR randomised clinical trial*[Text Word] OR randomized trial*[Text Word] OR randomised trial*[Text Word] OR "random allocation"[Text Word] OR allocated random*[Text Word] OR random assignment*[Text Word] OR "Clinical Trial, Phase III"[Publication Type] OR "Clinical Trial, Phase IV"[Publication Type])) |
| --- | --- |
| #14 | Search: ("Randomized Controlled Trial"[Publication Type] OR "randomized"[Title/Abstract] OR "randomised"[Title/Abstract] OR "randomization"[Title/Abstract] OR "randomisation"[Title/Abstract] OR "Clinical Trials, Phase III as Topic"[MeSH] OR "Clinical Trials, Phase IV as Topic"[MeSH] OR randomly[Title/Abstract] OR trial*[Title] OR "Randomized Controlled Trials as Topic"[MeSH] OR randomized controlled trial*[Text Word] OR randomised controlled trial*[Text Word] OR randomized clinical trial*[Text Word] OR randomised clinical trial*[Text Word] OR randomized trial*[Text Word] OR randomised trial*[Text Word] OR "random allocation"[Text Word] OR allocated random*[Text Word] OR random assignment*[Text Word] OR "Clinical Trial, Phase III"[Publication Type] OR "Clinical Trial, Phase IV"[Publication Type]) |
| #13 | Search: ((((((recovery of function[MeSH Terms]) OR (postoperative complications[MeSH Terms])) OR (length of stay[MeSH Terms])) OR (quality of life[MeSH Terms])) OR (postoperative recovery)) OR (complication rate)) OR (hospital stay) |
| #12 | Search: ((((prehabilitation) OR (prehab*)) OR (pre-hab*)) OR ((((preop*) OR (pre-op*)) OR (pre-surg*)) OR (presurg*))) AND (((((((exercise[MeSH Terms]) OR ("physical activity")) OR (fitness)) OR (training)) OR (jogging)) AND (((((nutrition[MeSH Terms]) OR (diet*)) OR (nutri*)) OR (intake)) OR (supple*))) AND (((((((drug therapy) OR (pharmacotherapy)) OR (medication)) OR (drug)) OR (pharmac*)) OR (medicinal)) OR (medicine))) |
| #11 | Search: ((((((exercise[MeSH Terms]) OR ("physical activity")) OR (fitness)) OR (training)) OR (jogging)) AND (((((nutrition[MeSH Terms]) OR (diet*)) OR (nutri*)) OR (intake)) OR (supple*))) AND (((((((drug therapy) OR (pharmacotherapy)) OR (medication)) OR (drug)) OR (pharmac*)) OR (medicinal)) OR (medicine)) |
| #10 | Search: ((((((drug therapy) OR (pharmacotherapy)) OR (medication)) OR (drug)) OR (pharmac*)) OR (medicinal)) OR (medicine) |
| #9 | Search: ((((nutrition[MeSH Terms]) OR (diet*)) OR (nutri*)) OR (intake)) OR (supple*) |
| #8 | Search: ((((exercise[MeSH Terms]) OR ("physical activity")) OR (fitness)) OR (training)) OR (jogging) |
| #7 | Search: (((prehabilitation) OR (prehab*)) OR (pre-hab*)) OR ((((preop*) OR (pre-op*)) OR (pre-surg*)) OR (presurg*)) |
| #6 | Search: (((preop*) OR (pre-op*)) OR (pre-surg*)) OR (presurg*) |
| #5 | Search: ((((surg*) OR (resection)) OR (operat*)) AND (((surg*) OR (resection)) OR (operat*)) AND ((((((((((((abdo*) OR (bowel)) OR (Intestin*)) OR (colo*)) OR (duodenum)) OR (jejunum)) OR (rectum)) OR (proct*)) OR (sigmoid)) OR (anus)) OR (anal)) OR (nephr*) OR ((((laparotomy[MeSH Terms]) OR (laparoscopy[MeSH Terms])) OR (abdominal surgery)) OR (laparoscopic surgery)) |
| #4 | Search: (((surg*) OR (resection)) OR (operat*)) AND ((((((((((((abdo*) OR (bowel)) OR (Intestin*)) OR (colo*)) OR (duodenum)) OR (jejunum)) OR (rectum)) OR (proct*)) OR (sigmoid)) OR (anus)) OR (anal)) OR (nephr*) |
| #3 | Search: ((((((((((((abdo*) OR (bowel)) OR (Intestin*)) OR (colo*)) OR (duodenum)) OR (jejunum)) OR (rectum)) OR (proct*)) OR (sigmoid)) OR (anus)) OR (anal)) OR (nephr*) |
| #2 | Search: ((surg*) OR (resection)) OR (operat*) |
| #1 | Search: (((laparotomy[MeSH Terms]) OR (laparoscopy[MeSH Terms])) OR (abdominal surgery)) OR (laparoscopic surgery) |

## Database: Ovid MEDLINE(R) <1946 to February 3 2025>

***Search Strategy:***

1. Laparotomy.mp
2. exp abdominal surgery/
3. exp colon/
4. exp rectum/
5. exp colorectal/
6. Colorectal Cancer.mp
7. exp Colorectal Neoplasms/
8. Prehabilitation.mp
9. exp Preoperative Care/
10. (aerobic exercise) OR (resistance training) OR (physical activity) OR (hik*) OR (high-speed resistance training) OR (multicomponent exercise program) OR (power training) OR (run*) OR (jog*) OR (cycl*) OR (walk*) OR (swim*) OR (activit*) OR (physical medicine).mp.
11. (nutrition) OR (diet*) OR (supple*) OR (intake).mp.
12. Postoperative Outcomes.mp
13. (complication) OR (length of day) OR (6-minute walking) OR (physical function) OR (psychology) OR (anxiety) OR (quality of life) .mp.
14. exp Treatment Outcome/
15. Randomized Controlled Trial.pt
16. exp Randomized Controlled Trials as Topic/
17. (random$ adj5 control$ adj5 trial$).mp.
18. (clinical trial$).mp
19. exp Clinical Trials as Topic/
20. (crossover or cross-over).mp.
21. randomi$.mp.
22. (random$ adj5 (assign$ or allocat$ or assort$ or receiv$)).mp.
23. 1 or 2 or 3 or 4 or 5 or 6 or 7
24. 8 or 9 or 10 or 11
25. 12 or 13 or 14
26. 15 or 16 or 17 or 18 or 19 or 20 or 21 or 22
27. 23 and 24 and 25 and 26

## Database: Embase <1974 to February 3 2025>

***Search Strategy:***

1. Laparotomy.mp
2. exp abdominal surgery/
3. exp colon/
4. exp rectum/
5. exp colorectal/
6. Colorectal Cancer.mp
7. exp Colorectal Neoplasms/
8. Prehabilitation.mp
9. exp Preoperative Care/
10. (aerobic exercise) OR (resistance training) OR (physical activity) OR (hik*) OR (high-speed resistance training) OR (multicomponent exercise program) OR (power training) OR (run*) OR (jog*) OR (cycl*) OR (walk*) OR (swim*) OR (activit*) OR (physical medicine).mp.
11. (nutrition) OR (diet*) OR (supple*) OR (intake).mp.
12. Postoperative Outcomes.mp
13. (complication) OR (length of day) OR (6-minute walking) OR (physical function) OR (psychology) OR (anxiety) OR (quality of life) .mp.
14. exp Treatment Outcome/
15. Randomized Controlled Trial.pt
16. exp Randomized Controlled Trials as Topic/
17. (random$ adj5 control$ adj5 trial$).mp.
18. (clinical trial$).mp
19. exp Clinical Trials as Topic/
20. (crossover or cross-over).mp.
21. randomi$.mp.
22. (random$ adj5 (assign$ or allocat$ or assort$ or receiv$)).mp.
23. 1 or 2 or 3 or 4 or 5 or 6 or 7
24. 8 or 9 or 10 or 11
25. 12 or 13 or 14
26. 15 or 16 or 17 or 18 or 19 or 20 or 21 or 22
27. 23 and 24 and 25 and 26

## Cochrane

#1 MeSH descriptor: [Laparotomy] explode all trees

#2 MeSH descriptor: [Colorectal Cancer] explode all trees

#3 MeSH descriptor: [Exercise] explode all trees

#4 MeSH descriptor: [Nutrition] explode all trees

#5 MeSH descriptor: [Prehabilitation] explode all trees

#6 MeSH descriptor: [Complication] explode all trees

#7 (aerobic exercise) OR (resistance training) OR (physical activity) OR (hik*) OR (high-speed resistance training) OR (multicomponent exercise program) OR (power training) OR (treadmill training) OR (whole body vibration) OR (run*) OR (jog*) OR (cycl*) OR (walk*) OR (swim*) OR (activit*) OR (physical medicine) OR (yoga) OR (Exercise Movement Techniques) in Trials (Word variations have been searched)

#8 ( abdominal surgery) OR (colon) OR (rectum) OR (colorectal) in Trials (Word variations have been searched)

#9 (complication) OR (length of day) OR (6-minute walking) OR (physical function) OR (psychology) OR (anxiety) OR (quality of life) in Trials (Word variations have been searched)

#10 #1 or #2 or #8

#11 #3 or #4 or #5 or #7

#12 #6 or #9

#13 #10 and #11 and #12

## Database: Web of Science <1965 to February 3 2025>

| # 5 | #1 AND #2 AND #3 AND #4  Indexes=SCI-EXPANDED, SSCI, A&HCI, CPCI-S, CPCI-SSH, BKCI-S, BKCI-SSH, ESCI, CCR-EXPANDED, IC Timespan=All years |  |  |
| --- | --- | --- | --- |
| # 3 | TOPIC: ((“randomized controlled trial*” or “controlled clinical trial” or “random*” or “clinical trial*” or randomly or trial or “clinical trial” or “randomized controlled trial*” or “cross-over studies” or clinic*) )  Indexes=SCI-EXPANDED, SSCI, A&HCI, CPCI-S, CPCI-SSH, BKCI-S, BKCI-SSH, ESCI, CCR-EXPANDED, IC Timespan=All years |  |  |
| # 3 | TOPIC: (“complication” or “length of day” or “6-minute walking” or “physical function” or “psychology” or “anxiety” or “quality of life”)  Indexes=SCI-EXPANDED, SSCI, A&HCI, CPCI-S, CPCI-SSH, BKCI-S, BKCI-SSH, ESCI, CCR-EXPANDED, IC Timespan=All years |  |  |
| # 2 | TOPIC: (“Exercise” or “Nutrition” or “Prehabilitation” or “aerobic exercise” or “resistance training” or “physical activity” or “high-speed resistance training” or “multicomponent exercise program” or “power training” or “treadmill training” or “whole body vibration” or “run*” or “jog*” or “cycl*” or “walk*” or “swim*” or “activit*” or “physical medicine” or “yoga” or “Exercise Movement Techniques” in Trials (Word variations have been searched)  Indexes=SCI-EXPANDED, SSCI, A&HCI, CPCI-S, CPCI-SSH, BKCI-S, BKCI-SSH, ESCI, CCR-EXPANDED, IC Timespan=All years |  |  |
| # 1 | TOPIC: ("Laparotomy" or "Colorectal Cancer" or "abdominal surgery" or "colon" or "rectum" or "colorectal")  Indexes=SCI-EXPANDED, SSCI, A&HCI, CPCI-S, CPCI-SSH, BKCI-S, BKCI-SSH, ESCI, CCR-EXPANDED, IC Timespan=All years |  |  |

# Supplementary 2: Assessment of the transitivity

Different clinical trials need to ensure that their baseline levels are consistent. If the baseline levels are inconsistent, the results cannot be transitive. Therefore, the transitivity assumption was evaluated by comparing the distribution of potential effect modifiers (laparoscopic surgery proportion, sample size, mean age, percentage female, prehabilitation duration) across studies grouped before analyzing the results, and we use the R ggplot2 package to draw boxplots between the above potential influencing factors and various types of prehabilitation.

## 2.1 laparoscopic surgery proportion

We checked the laparoscopic surgery proportion of the included studies. The range is from 8.0% to 100%, with a median of 80%. The results of one-way ANOVA showed that there was not statistical difference in the laparoscopic surgery proportion between the types of prehabilitation (F = 0.294, P = 0.829).

******

**Figure 2.1:** Boxplot for distribution of laparoscopic surgery proportion. BT breath training, CON standard care, EX exercise, Mul multimodal prehabilitation programme (exercise, nutritional, and anxiety-reduction strategies), NU nutritional intervention.

## 2.2 Mean age

We checked the mean age distribution of the included study participants. The range is from 53.86 to 83.5, with a median of 67.6. The results of one-way ANOVA showed that there was no statistical difference in the mean age between the types of prehabilitation (F = 0.289, P = 0.833).

******

**Figure 2.2:** Boxplot for distribution of mean age. BT breath training, CON standard care, EX exercise, Mul multimodal prehabilitation programme (exercise, nutritional, and anxiety-reduction strategies), NU nutritional intervention.

## 2.3 Percentage female

We checked the percentage female distribution of the included study participants. The range is from 0% to 79.2%, with a median of 40.1%. The results of one-way ANOVA showed that there was no statistical difference in the percentage female between the types of prehabilitation (F = 0.258, P = 0.856).

**Figure 2.3:** Boxplot for distribution of percentage female. BT breath training, CON standard care, EX exercise, Mul multimodal prehabilitation programme (exercise, nutritional, and anxiety-reduction strategies), NU nutritional intervention.

## 2.4 Sample size

We checked the sample size distribution of the included studies each arm. The range is from 7 to 351, with a median of 40. The results of one-way ANOVA showed that there was no statistical difference in the percentage female between the types of prehabilitation (F = 0.17, P = 0.916).

**Figure 2.4:** Boxplot for distribution of sample size. BT breath training, CON standard care, EX exercise, Mul multimodal prehabilitation programme (exercise, nutritional, and anxiety-reduction strategies), NU nutritional intervention.

## 2.5 Sample size

We checked the prehabilitation duration distribution of the included studies each arm. The range is from 1 to 10, with a median of 4. The results of one-way ANOVA showed that there was no statistical difference in the percentage female between the types of prehabilitation (F = 0.113, P = 0.952).

**Figure 2.5:** Boxplot for distribution of prehabilitation duration. BT breath training, CON standard care, EX exercise, Mul multimodal prehabilitation programme (exercise, nutritional, and anxiety-reduction strategies), NU nutritional intervention.

# Supplementary 3: Characteristics of studies and subjects included in the review

## Table 3.1: Characteristics of subjects included in the review

| **Study** | **Surgical procedure**  **(prehab/ control)** | **Subjects**  **(prehab/ control)** | **Sex (m/f)**  **(prehab vs. control)** | **Mean age**  **(prehab vs. control)** |
| --- | --- | --- | --- | --- |
| Awasthi et al. (2019) | Laparoscopic: 20-25% | 140 (63/77) | 46/17 vs. 48/29 | 69.9±10.7 vs. 65.9±11.4 |
| Bao (2023) | N/A | 86 (43/43) | 26/17 vs. 25/18 | 67.75±8.27 vs. 68.12±8.43 |
| Barberan-Garcia et al. (2018) | Laparoscopic: 79%/89% | 125 (62/63) | 43/19 vs. 51/12 | 71±11 vs. 71±10 |
| Berkel et al. (2022) | Laparoscopic: 82%/72% | 57 (28/29) | 16/12 vs. 14/15 | 74±7 vs. 73±6 |
| Boden et al. (2018) | N/A | 181 (92/89) | N/A | 63.4±15.1 vs. 67.5±14.1 |
| Bousquet-Dion et al. (2018) | Laparoscopic: 84%/81% | 63 (37/26) | 30/7 vs. 16/10 | 74±7.8 vs. 71±14.8 |
| F. Carli et al. (2010) | Laparoscopic: 24%/24% | 112 (58/54) | 34/24 vs. 31/23 | 61±16 vs. 60±15 |
| Francesco Carli et al. (2020) | Laparoscopic: 76.4%/81.8% | 110 (55/55) | 29/26 vs. 23/32 | 78±7.4 vs. 82±6.7 |
| Fulop et al. (2021) | N/A | 149 (77/72) | 40/37 vs. 33/39 | 70±11.1 vs. 70±8.1 |
| Gillis et al. (2016) | Laparoscopic: 90%/75% | 43 (22/21) | 15/7 vs. 13/8 | 67.6±11.5 vs. 69.1±9.4 |
| Karlsson et al. (2019) | Laparoscopic: 70%/8% | 21 (10/11) | 4/6 vs. 4/7 | 83.5±6.7 vs. 74.0±2.2 |
| Kim et al. (2009) | N/A | 21 (14/7) | 9/5 vs. 4/3 | 55±15 vs. 65±9 |
| Li (2023) | N/A | 80 (40/40) | 23/17 vs. 24/16 | 53.86±10.24 vs. 53.27±10.11 |
| López-Rodríguez-Arias et al. (2021) | N/A | 20 (10/10) | 4/6 vs. 3/7 | 66.5±10 vs. 66±8 |
| MacFie et al. (2000) | N/A | 49 (24/25) | 15/19 vs. 12/13 | 68±15.2 vs. 64±10.75 |
| Molenaar et al. (2023) | Laparoscopic: 95.9%/90.6% | 251 (123/128) | 62/61 vs. 76/52 | 69±12.6 vs. 71±11.9 |
| Moug et al. (2019) | Laparoscopic: 36%/21% | 48 (24/24) | 18/6 vs.13/11 | 65.2±11 vs. 66.5±10 |
| Northgraves et al. (2020) | Laparoscopic: 40%/36.4% | 21 (10/11) | 7/4 vs. 4/6 | 64.1±10.5 vs. 63.5±12.5 |
| Onerup et al. (2022) | Laparoscopic: 56%/52% | 668 (317/351) | 190/127 vs. 210/141 | 69±11 vs. 68±11 |
| Parker et al. (1985) | Laparoscopic: 97%/90% | 77 (38/39) | 21/17 vs. 27/12 | 65.7±13.6 vs. 66.0±9.1 |
| Qin et al. (2021) | Laparoscopic: 100% | 200 (100/100) | 60/40 vs. 62/38 | 63.9±12.6 vs. 63.7±12.6 |
| Smedley et al. (2004) | N/A | 98 (48/50) | 33/15 vs. 28/22 | 61±15.3 vs. 63±15.8 |
| Triguero-Cánovas et al. (2023) | N/A | 44 (23/21) | 16/7 vs. 13/8 | 68.1±7.7 vs. 67.2±8.5 |
| Waller et al. (2022) | N/A | 22 (11/11) | 4/7 vs. 7/4 | 55.5±9.3 vs. 61.0±11.8 |
| Xu et al. (2023) | Laparoscopic: 100% | 80 (40/40) | 31/9 vs. 30/10 | 58.50±2.13 vs. 58.20±2.15 |
| Yan (2021) | Laparoscopic: 100% | 80 (40/40) | 28/12 vs. 27/13 | 67.56±4.09 vs. 67.25±4.03 |
| Zhang et al. (2023) | N/A | 100 (50/50) | 29/21 vs. 30/20 | 69.63±5.24 vs. 70.02±5.21 |

N/A None available, prehab prehabilitation, m male, f female.

## Table 3.2 Characteristics of prehabilitation preogramme included in the review

| **Study** | **Intervention detail** | | **Training frequency** | **Session duration** | **Intensity/control of intensity** | **Duration** | **Overall training sessions** | **Adherence in training sessions** |
| --- | --- | --- | --- | --- | --- | --- | --- | --- |
|  | **Prehabilitation group** | **Control group** |  |  |  |  |  |  |
| Awasthi et al. (2019) | Mul: (a) home-based whole-body exercise (aerobic and resistance training;  (30 min)  (b) supervised stepper and resistance training (60 min)  (c) nutritional intervention  (d) anxiety-reduction strategies | Standard care | (a) 3–4 × per week (b) 1 × per week | (a) 30 min  (b) 60 min | (a) 60–70% of maximum heart rate  (b) Borg scale > 12 | N/A | N/A | 98% |
| Bao (2023) | Mul: (a) Telephone-guided aerobic exercise (10-30 min)  (b) anaerobic exercise (10-15 min)  (c) inspiratory muscle training (10 min)  (d) nutrition intervention  (e) psychosocial support | Standard care | (a) 3 × per week  (b) 3 × per week  (c) 3 × per day | (a) 10-30 min  (b) 10-15 min  (c) 10 min | Maximum heart rate (70-80%, weekly increase) | N/A | N/A | N/A |
| Barberan-Garcia et al. (2018) | EX: (a) Non-supervised home- and step-based physical activity  (b) supervised high interval endurance training and resistance training (personalized and progressive bicycle ergometer; 40 min) | Standard care | (a) Daily  (b) 1-3 × per week | (b) 40 min | (a) steps per day  (b) Interval 70–85% max work rate rest 40% max work rate and Borg scale | (b) 12±5 | 6±2 weeks | N/A |
| Berkel et al. (2022) | EX: (a) supervised moderate to high intensity interval training (cycle ergometer; 40 min)  (b) supervised resistance training (20 min) | Standard care | 3 × per week | (a) 40 min  (b) 20 min | (a) intervals 120% of ventilatory threshold and adapting due to Borg scale  (b) 70–80% of One repetition maximum | 8.1±2.1 | 3 weeks | 90% |
| Boden et al. (2018) | BT: Breathing exercise training (30 min) | Standard care | N/A |  | N/A | N/A | 6 weeks | N/A |
| Bousquet-Dion et al. (2018) | Mul: (a) home-based whole-body exercise (aerobic and resistance training;  (30 min)  (b) supervised stepper and resistance training (60 min)  (c) nutritional intervention  (d) anxiety-reduction strategies | Standard care | (a) 3–4 × per week  (b) 1 × per week | (a) 30 min  (b) 60 min | (a) 60–70% of maximum heart rate  (b) Borg scale > 12 | N/A | N/A | 98% |
| F. Carli et al. (2010) | EX: (a) home-based aerobic cycling exercise (20–30 min)  (b) home-based resistance training (15 min) | Mul: home-based walking and breathing training (30 min) | Daily | (a) 20–30 min  (b) 15 min | Maximum heart rate (50%, weekly increase) | 8.3±6.2 h | 52 days | N/A |
| Francesco Carli et al. (2020) | Mul: (a) supervised moderate aerobic stepper  training (30 min) and resistance training (25 min)  (b) personalized home-based aerobic activities daily and resistance training (30 min)  (c) nutrition intervention  (d) psychological intervention | Standard care | (a) 1 × per week  (b) walking activity daily and 3 × per week resistance training | (a) 25 min  (b) 30 min | N/A | N/A | 4 weeks | 68% |
| Fulop et al. (2021) | Mul: (a) home-based aerobic and breathing training (30 min)  (b) nutrition  (c) psychological intervention | Standard care | Daily | 30 min | According patient’s ability | N/A | 4 weeks | N/A |
| Gillis et al. (2016) | NU: Whey protein supplementation (1.2-1.5g protein/kg/d) | Standard care | Daily | N/A | N/A | N/A | 4 weeks | N/A |
| Karlsson et al. (2019) | EX: Supervised home-based training (60 min) including inspiratory muscle training, high-intensity functional strength training and endurance training | Standard care | 2-3 × per week | 60 min | Borg scale (CR-10) (7–8) | N/A | 2-3 weeks | 97% |
| Kim et al. (2009) | EX: Home-based aerobic cycle ergometer training (20–30 min) | Standard care | Daily | 20-30 min | %HRR (40–65% and Borg Scale (12–16) | 27±9 | 3 weeks | 74% |
| Li (2023) | Mul: (a) inspiratory muscle training (15-20 min)  (b) Supervised aerobic and resistance training  (c) nutrition intervention  (d) psychosocial interventions | Standard care | (a) 3 × per day | (a) 15-20 min | According patient’s ability | N/A | 4.50±1.23 days | N/A |
| López-Rodríguez-Arias et al. (2021) | Mul: (a) home based aerobic, resistance video training and breathing exercise  (b) Nutrition  (c) coping strategies to reduce anxiety | Standard care | Daily | N/A | N/A | N/A | 4 weeks | N/A |
| MacFie et al. (2000) | NU: Supplements containing protein and carbohydrates in addition to diet. | Standard care | Daily | N/A | N/A | N/A | N/A | N/A |
| Molenaar et al. (2023) | Mul: (a) Supervised aerobic and resistance training (60 min)  (b) nutrition intervention  (c) anxiety-coping interventions | Standard care | 3 × per week | 60 min | Aerobic with 85-90% peakpower (4 times) and 30% peakpower (4 times);  Resistance exercise with 65-70% 1RM | N/A | 4 weeks | N/A |
| Moug et al. (2019) | EX:Telephone-guided walking programme | Standard care | Daily | N/A | N/A | N/A | 13-17 weeks | N/A |
| Northgraves et al. (2020) | EX: Individualized aerobic (25 min) and functional resistance training (25 min) | Standard care | 3 × per week | 50 min | HRR (40–60%) and Borg scale (11–13) | 6.9 ± 2.3 | 3 weeks | 89.60% |
| Onerup et al. (2022) | EX: home based individualized aerobic activity (30 min); inspiratory muscle training | Standard care | Daily | 30 min | Borg scale (medium intensity) | N/A | 2 weeks | N/A |
| Parker et al. (1985) | Mul: (a) home-based unsupervised aerobic and  resistance training (50 min)  (b) nutrition intervention  (c) coping strategies to reduce anxiety | Standard care | At least 3 × per week | 50 min | Bore scale > 12 | N/A | 4 weeks | 78% |
| Qin et al. (2021) | EX: (a) Supervised inspiratory muscle training (5-10 min)  (b) upper exercise (5min)  (c) lower exercise (5-10 min) | Standard care | (a) 2 × per day  (b) 2 × per day  (c) 3 × per day | (a) 5-10 min  (b) 5 min  (c) 5-10 min | N/A | N/A | > 5 days | N/A |
| Smedley et al. (2004) | NU: Protein Oral Nutritional Supplementation | Standard care | Daily | N/A | N/A | N/A | N/A | N/A |
| Triguero-Cánovas et al. (2023) | Mul: (a) home based aerobic exercise (30-50 min)  (b) homebased resistance exercise (20 min)  (c) nutrition intervention  (d) Suggestions for relaxation and breathing exercises | Standard care | (a) Daily  (b) 3 × per week | (a) 30-50 min  (b) 20 min | Respiratory exchange rate > 1.05 | N/A | N/A | N/A |
| Waller et al. (2022) | Mul: (a) home based aerobic exercise (30 min)  (b) homebased resistance exercise (band consisting, 30 min)  (c) nutrition  (d) psychosocial support | Standard care | (a) 3 × per week  (b) 2 × per week | (a) 30 min  (b) 30 min | HR (50–70% of maxi- mum heart rate) and Borg scale (12–16) | N/A | > 2 weeks | 84% |
| Xu et al. (2023) | Mul: (a) Supervised exercise  (b) nutrition  (c) psychology | Standard care | N/A | N/A | N/A | N/A | N/A | N/A |
| Yan (2021) | Mul: (a) Supervised aerobic and resistance training (40-60 min)  (b) nutrition intervention  (c) psychosocial interventions | Standard care | 3-5 × per week | 40-60 min | N/A | N/A | 4-8 weeks | N/A |
| Zhang et al. (2023) | Mul: (a) Supervised aerobic, resistance training and breathing exercise (50 min)  (b) nutrition intervention  (c) anxiety-reduction strategies | Standard care | Daily | 50 min | aerobic, walking about 7,500 steps; resistance training, 5kg grip; breathing exercise, blowing up a balloon to 20cm; | N/A | N/A | N/A |

N/A None available, BT breath training, CON standard care, EX exercise, Mul multimodal prehabilitation programme (exercise, nutritional, and anxiety-reduction strategies), NU nutritional intervention.

## Table 3.3 Characteristics of outcomes included in the review

| **Study** | **6MWT** | **Postoperative complications** | **Hospital days of stay** | **Anxiety** | **Depression** |
| --- | --- | --- | --- | --- | --- |
| Awasthi et al. (2019) | Presurgery, and postsurgery 4 and 8 weeks, 6MWT, m | N/A | N/A | Presurgery, and postsurgery 4 and 8 weeks HAD anxiety | Presurgery, and postsurgery 4 and 8 weeks HAD depression |
| Bao (2023) | N/A | N/A | Hospital days of stay | N/A | N/A |
| Barberan-Garcia et al. (2018) | Presurgery, 6MWT, m | Postoperative complications | Hospital days of stay | Presurgery, HAD anxiety | Presurgery, HAD depression |
| Berkel et al. (2022) | N/A | Postoperative complications | Hospital days of stay | N/A | N/A |
| Boden et al. (2018) | N/A | Postoperative complications | Hospital days of stay | N/A | N/A |
| Bousquet-Dion et al. (2018) | Presurgery, and postsurgery 4 and 8 weeks, 6MWT, m | Postoperative complications | Hospital days of stay | N/A | N/A |
| F. Carli et al. (2010) | Presurgery, and postsurgery 10 weeks, 6MWT, m | Postoperative complications | Hospital days of stay | Presurgery, and postsurgery 10 weeks HAD anxiety | Presurgery, , and postsurgery 10 weeks HAD depression |
| Francesco Carli et al. (2020) | Presurgery, and postsurgery 4 weeks, 6MWT, m | Postoperative complications | Hospital days of stay | Presurgery, and postsurgery 4 weeks HAD anxiety | Presurgery, , and postsurgery 4 weeks HAD depression |
| Fulop et al. (2021) | Presurgery, and postsurgery 4 and 8 weeks, 6MWT, m | N/A | Hospital days of stay | Presurgery, and postsurgery 4 and 8 weeks HAD anxiety | Presurgery, , and postsurgery 4 and 8 weeks HAD depression |
| Gillis et al. (2016) | Presurgery, and postsurgery 4 weeks, 6MWT, m | Postoperative complications | Hospital days of stay | N/A | N/A |
| Karlsson et al. (2019) | Presurgery, and postsurgery 4 weeks, 6MWT, m | Postoperative complications | Hospital days of stay | N/A | N/A |
| Kim et al. (2009) | Presurgery, 6MWT, m | N/A | N/A | N/A | N/A |
| Li (2023) | N/A | Postoperative complications | Hospital days of stay | N/A | N/A |
| López-Rodríguez-Arias et al. (2021) | N/A | Postoperative complications | Hospital days of stay | N/A | N/A |
| MacFie et al. (2000) | N/A | Postoperative complications | Hospital days of stay | N/A | N/A |
| Molenaar et al. (2023) | Postsurgery 4 weeks, 6MWT, m | Postoperative complications | Hospital days of stay | N/A | N/A |
| Moug et al. (2019) | Presurgery, 6MWT, m | Postoperative complications | Hospital days of stay | N/A | Presurgery, BDI-Ⅱ |
| Northgraves et al. (2020) | Presurgery, 6MWT, m | N/A | N/A | N/A | N/A |
| Onerup et al. (2022) | N/A | N/A | N/A | N/A | N/A |
| Parker et al. (1985) | Presurgery, and postsurgery 8 weeks, 6MWT, m | Postoperative complications | Hospital days of stay | Presurgery, and postsurgery 4 and 8 weeks HAD anxiety | Presurgery, and postsurgery 4 and 8 weeks HAD depression |
| Qin et al. (2021) | N/A | Postoperative complications | Hospital days of stay | N/A | N/A |
| Smedley et al. (2004) | N/A | Postoperative complications | Hospital days of stay | N/A | N/A |
| Triguero-Cánovas et al. (2023) | Postsurgery 45 days, 6MWT, m | Postoperative complications | Hospital days of stay | N/A | N/A |
| Waller et al. (2022) | Presurgery, 6MWT, m | N/A | N/A | Presurgery HAD anxiety | Presurgery HAD depression |
| Xu et al. (2023) | Presurgery, and postsurgery 4 weeks, 6MWT, m | Postoperative complications | Hospital days of stay | Presurgery, and postsurgery 4 weeks HAD anxiety | Presurgery, , and postsurgery 4 weeks HAD depression |
| Yan (2021) | Postsurgery 1 month, 6MWT, m | Postoperative complications | Hospital days of stay | N/A | N/A |
| Zhang et al. (2023) | Presurgery, and discharged after surgery, 6MWT, m | Postoperative complications | Hospital days of stay | N/A | N/A |

N/A None available, 6 MWT, 6 minutes walk test, HAD Hospital Anxiety and Depression Scale, BDI Beck's Depression Inventory.

**List of included studies:**

Awasthi, R., Minnella, E. M., Ferreira, V., Ramanakumar, A. V., Scheede-Bergdahl, C., & Carli, F. (2019). Supervised exercise training with multimodal pre-habilitation leads to earlier functional recovery following colorectal cancer resection. *Acta Anaesthesiologica Scandinavica, 63*(4), 461-467. doi:10.1111/aas.13292

Bao, Y. (2023). Effect of preoperative pre-rehabilitation nursing on postoperative nutritional status and psychological state of patients with colorectal cancer. *Modern Nurse, 30*(09), 50-53. doi:10.19791/j.cnki.1006-6411.2023.25.014

Barberan-Garcia, A., Ubré, M., Roca, J., Lacy, A. M., Burgos, F., Risco, R., . . . Martínez-Pallí, G. (2018). Personalised Prehabilitation in High-risk Patients Undergoing Elective Major Abdominal Surgery: A Randomized Blinded Controlled Trial. *Annals of Surgery, 267*(1), 50-56. doi:10.1097/SLA.0000000000002293

Berkel, A. E. M., Bongers, B. C., Kotte, H., Weltevreden, P., de Jongh, F. H. C., Eijsvogel, M. M. M., . . . Klaase, J. M. (2022). Effects of Community-based Exercise Prehabilitation for Patients Scheduled for Colorectal Surgery With High Risk for Postoperative Complications: Results of a Randomized Clinical Trial. *Annals of Surgery, 275*(2), e299-e306. doi:10.1097/SLA.0000000000004702

Boden, I., Skinner, E. H., Browning, L., Reeve, J., Anderson, L., Hill, C., . . . Denehy, L. (2018). Preoperative physiotherapy for the prevention of respiratory complications after upper abdominal surgery: pragmatic, double blinded, multicentre randomised controlled trial. *BMJ (Clinical Research ed.), 360*, j5916. doi:10.1136/bmj.j5916

Bousquet-Dion, G., Awasthi, R., Loiselle, S.-È., Minnella, E. M., Agnihotram, R. V., Bergdahl, A., . . . Scheede-Bergdahl, C. (2018). Evaluation of supervised multimodal prehabilitation programme in cancer patients undergoing colorectal resection: a randomized control trial. *Acta Oncologica (Stockholm, Sweden), 57*(6), 849-859. doi:10.1080/0284186X.2017.1423180

Carli, F., Bousquet-Dion, G., Awasthi, R., Elsherbini, N., Liberman, S., Boutros, M., . . . Fiore, J. F. (2020). Effect of Multimodal Prehabilitation vs Postoperative Rehabilitation on 30-Day Postoperative Complications for Frail Patients Undergoing Resection of Colorectal Cancer: A Randomized Clinical Trial. *JAMA Surgery, 155*(3), 233-242. doi:10.1001/jamasurg.2019.5474

Carli, F., Charlebois, P., Stein, B., Feldman, L., Zavorsky, G., Kim, D. J., . . . Mayo, N. E. (2010). Randomized clinical trial of prehabilitation in colorectal surgery. *The British Journal of Surgery, 97*(8), 1187-1197. doi:10.1002/bjs.7102

Fulop, A., Lakatos, L., Susztak, N., Szijarto, A., & Banky, B. (2021). The effect of trimodal prehabilitation on the physical and psychological health of patients undergoing colorectal surgery: a randomised clinical trial. *Anaesthesia, 76*(1), 82-90. doi:10.1111/anae.15215

Gillis, C., Loiselle, S.-E., Fiore, J. F., Awasthi, R., Wykes, L., Liberman, A. S., . . . Carli, F. (2016). Prehabilitation with Whey Protein Supplementation on Perioperative Functional Exercise Capacity in Patients Undergoing Colorectal Resection for Cancer: A Pilot Double-Blinded Randomized Placebo-Controlled Trial. *Journal of the Academy of Nutrition and Dietetics, 116*(5), 802-812. doi:10.1016/j.jand.2015.06.007

Karlsson, E., Farahnak, P., Franzén, E., Nygren-Bonnier, M., Dronkers, J., van Meeteren, N., & Rydwik, E. (2019). Feasibility of preoperative supervised home-based exercise in older adults undergoing colorectal cancer surgery - A randomized controlled design. *PloS One, 14*(7), e0219158. doi:10.1371/journal.pone.0219158

Kim, D. J., Mayo, N. E., Carli, F., Montgomery, D. L., & Zavorsky, G. S. (2009). Responsive measures to prehabilitation in patients undergoing bowel resection surgery. *The Tohoku Journal of Experimental Medicine, 217*(2), 109-115. Retrieved from https://pubmed.ncbi.nlm.nih.gov/19212103

Li, B. (2023). Effect of nursing intervention based on prehabilitation concept on complications and length of stay in surgical patients with colorectal cancer and hypertension. *Prevention and Treatment of Cardiovascular Disease, 13*(17), 43-45.

López-Rodríguez-Arias, F., Sánchez-Guillén, L., Aranaz-Ostáriz, V., Triguero-Cánovas, D., Lario-Pérez, S., Barber-Valles, X., . . . Arroyo, A. (2021). Effect of home-based prehabilitation in an enhanced recovery after surgery program for patients undergoing colorectal cancer surgery during the COVID-19 pandemic. *Supportive Care In Cancer : Official Journal of the Multinational Association of Supportive Care In Cancer, 29*(12), 7785-7791. doi:10.1007/s00520-021-06343-1

MacFie, J., Woodcock, N. P., Palmer, M. D., Walker, A., Townsend, S., & Mitchell, C. J. (2000). Oral dietary supplements in pre- and postoperative surgical patients: a prospective and randomized clinical trial. *Nutrition (Burbank, Los Angeles County, Calif.), 16*(9), 723-728. Retrieved from https://pubmed.ncbi.nlm.nih.gov/10978851

Molenaar, C. J. L., Minnella, E. M., Coca-Martinez, M., Ten Cate, D. W. G., Regis, M., Awasthi, R., . . . Slooter, G. D. (2023). Effect of Multimodal Prehabilitation on Reducing Postoperative Complications and Enhancing Functional Capacity Following Colorectal Cancer Surgery: The PREHAB Randomized Clinical Trial. *JAMA Surgery, 158*(6), 572-581. doi:10.1001/jamasurg.2023.0198

Moug, S. J., Mutrie, N., Barry, S. J. E., Mackay, G., Steele, R. J. C., Boachie, C., . . . Anderson, A. S. (2019). Prehabilitation is feasible in patients with rectal cancer undergoing neoadjuvant chemoradiotherapy and may minimize physical deterioration: results from the REx trial. *Colorectal Disease : the Official Journal of the Association of Coloproctology of Great Britain and Ireland, 21*(5), 548-562. doi:10.1111/codi.14560

Northgraves, M. J., Arunachalam, L., Madden, L. A., Marshall, P., Hartley, J. E., MacFie, J., & Vince, R. V. (2020). Feasibility of a novel exercise prehabilitation programme in patients scheduled for elective colorectal surgery: a feasibility randomised controlled trial. *Supportive Care In Cancer : Official Journal of the Multinational Association of Supportive Care In Cancer, 28*(7), 3197-3206. doi:10.1007/s00520-019-05098-0

Onerup, A., Andersson, J., Angenete, E., Bock, D., Börjesson, M., Ehrencrona, C., . . . Haglind, E. (2022). Effect of Short-term Homebased Pre- and Postoperative Exercise on Recovery After Colorectal Cancer Surgery (PHYSSURG-C): A Randomized Clinical Trial. *Annals of Surgery, 275*(3), 448-455. doi:10.1097/SLA.0000000000004901

Parker, M. C., Ashby, E. C., Nicholls, M. W., Dowding, C. H., & Brookes, J. C. (1985). Povidone-iodine bowel irrigation before resection of colorectal carcinoma. *Annals of the Royal College of Surgeons of England, 67*(4), 227-228. Retrieved from https://pubmed.ncbi.nlm.nih.gov/4037631

Qin, P., Jin, J., Min, S., Wang, W., & Chen, J. (2021). Effect of preoperative enhance recovery exercise on postoperative outcome in patients undergoing laparoscopic colorectal cancer surgery. *J Clin Anesthesiol, 37*(02), 119-122.

Smedley, F., Bowling, T., James, M., Stokes, E., Goodger, C., O'Connor, O., . . . Silk, D. (2004). Randomized clinical trial of the effects of preoperative and postoperative oral nutritional supplements on clinical course and cost of care. *The British Journal of Surgery, 91*(8), 983-990. Retrieved from https://pubmed.ncbi.nlm.nih.gov/15286958

Triguero-Cánovas, D., López-Rodríguez-Arias, F., Gómez-Martínez, M., Sánchez-Guillén, L., Peris-Castelló, F., Alcaide-Quirós, M. J., . . . Ramírez, J. M. (2023). Home-based prehabilitation improves physical conditions measured by ergospirometry and 6MWT in colorectal cancer patients: a randomized controlled pilot study. *Supportive Care In Cancer : Official Journal of the Multinational Association of Supportive Care In Cancer, 31*(12), 673. doi:10.1007/s00520-023-08140-4

Waller, E., Sutton, P., Rahman, S., Allen, J., Saxton, J., & Aziz, O. (2022). Prehabilitation with wearables versus standard of care before major abdominal cancer surgery: a randomised controlled pilot study (trial registration: NCT04047524). *Surgical Endoscopy, 36*(2), 1008-1017. doi:10.1007/s00464-021-08365-6

Xu, J., & Guan, S. (2023). Application of pre-rehabilitation nursing in the perioperative period of laparoscopic colorectal cancer surgery. *Zhejiang J Trauma Surg, 28*(03), 599-601.

Yan, Y. (2021). Application of preoperative prehabilitation in laparoscopic colorectal cancer surgery in the elderly. *Chinese Journal of Gerontology, 41*(11), 2298-2300.

Zhang, Y., Zhai, J., & Li, X. (2023). The Eficacy of"3-Union Advance Tactics about Rehabilitation" Preoperatively Applied in Senior Patients with Colorectal Cancer. *Chin J Coloproctol 43*(02), 59-61.

# Supplementary 4: Risk of Bias

## Table 4.1 The risk of bias assessment for the individual included studies

| **Study** | Randomization process | Deviations from intended interventions | Missing outcome data | Measurement of the outcome | Selection of the reported result | Overall Bias |
| --- | --- | --- | --- | --- | --- | --- |
| Awasthi et al. (2019) | Low | Some concerns | Low | Low | Low | Some concerns |
| Bao (2023) | Some concerns | Low | Low | Low | Low | Some concerns |
| Barberan-Garcia et al. (2018) | Some concerns | Low | Low | Low | Low | Some concerns |
| Berkel et al. (2022) | Low | Low | Low | Low | Low | Low |
| Boden et al. (2018) | Low | Low | Low | Low | Some concerns | Some concerns |
| Bousquet-Dion et al. (2018) | Low | Low | Low | Low | Low | Low |
| F. Carli et al. (2010) | Low | Low | Low | Low | Low | Low |
| Francesco Carli et al. (2020) | Some concerns | Low | Low | Low | Low | Some concerns |
| Fulop et al. (2021) | Low | Low | Some concerns | Low | Low | Some concerns |
| Gillis et al. (2016) | Low | Low | Low | Low | Low | Low |
| Karlsson et al. (2019) | Low | Low | Low | Low | Low | Low |
| Kim et al. (2009) | Some concerns | Some concerns | Some concerns | Low | Low | Some concerns |
| Li (2023) | Low | Low | High | Low | Low | High |
| López-Rodríguez-Arias et al. (2021) | Low | Low | Low | Low | Low | Low |
| MacFie et al. (2000) | Some concerns | Low | Low | Low | Low | Some concerns |
| Molenaar et al. (2023) | Low | Low | Low | Low | Low | Low |
| Moug et al. (2019) | Low | Low | Low | Low | Low | Low |
| Northgraves et al. (2020) | Low | Low | Low | Low | Low | Low |
| Onerup et al. (2022) | Low | Low | Some concerns | Low | Low | Some concerns |
| Parker et al. (1985) | Low | Low | Low | Low | Low | Low |
| Qin et al. (2021) | Low | Low | Low | Low | Low | Low |
| Smedley et al. (2004) | Low | Low | Low | Low | Low | Low |
| Triguero-Cánovas et al. (2023) | Low | Some concerns | Low | Some concerns | Low | Some concerns |
| Waller et al. (2022) | Low | Low | Low | Low | Low | Low |
| Xu et al. (2023) | Low | Low | High | Low | Low | High |
| Yan (2021) | Low | Low | Low | Low | Some concerns | Some concerns |
| Zhang et al. (2023) | Low | Low | Low | Low | Low | Low |

# Supplementary 5: Evaluation of heterogeneity and inconsistency

**Table 5.1: Quantifying heterogeneity**

| **Outcomes** | **τ^2^** | **Q** | **df** | **P** | **I^2^** | **Heterogeneity assessment** |
| --- | --- | --- | --- | --- | --- | --- |
| Postoperative complications | 0.6093 | 45.53 | 16 | <0.0001 | 64.9% | Moderate to high |
| Hospital day | 0.8462 | 98.29 | 18 | <0.0001 | 81.7% | High |
| 6 minutes walk test (Presurgery) | 197.3036 | 19.46 | 12 | 0.0781 | 38.3% | Low |
| 6 minutes walk test (Postsurgery 4 weeks) | 159.7659 | 15.03 | 7 | 0.0357 | 53.4% | Moderate |
| 6 minutes walk test (Postsurgery 8 weeks) | 771.4839 | 10.77 | 4 | 0.0293 | 62.9% | Moderate to high |
| Anxiety (Presurgery) | 0.6402 | 83.90 | 6 | <0.0001 | 92.8% | High |
| Anxiety (Postsurgery 4 weeks) | 0.9171 | 33.63 | 2 | <0.0001 | 94.1% | High |
| Anxiety (Postsurgery 8 weeks) | 0 | 0.47 | 2 | 0.7921 | 0% | Low |
| Depressive (Presurgery) | 0.3305 | 53.02 | 7 | <0.0001 | 86.8% | High |
| Depressive (Postsurgery 4 weeks) | 1.4783 | 48.40 | 2 | <0.0001 | 95.9% | High |
| Depressive (Postsurgery 8 weeks) | 0.0139 | 2.62 | 2 | 0.2702 | 23.6% | Low |

**Evaluation of inconsistency**

**Table 5.2 Summary of the global inconsistency and SIDE splitting results**

| Outcomes | the Design-by-Treatment test | | | |
| --- | --- | --- | --- | --- |
|  | Q | df | τ^2^ | p-value |
| Postoperative complications | 0.08 | 1 | 0.6940 | 0.7722 |
| Hospital day | 0.87 | 1 | 0.8473 | 0.3510 |
| 6 minutes walk test (Presurgery) | 0.13 | 1 | 262.7353 | 0.7204 |
| 6 minutes walk test (Postsurgery 4 weeks) | 0.00 | 0 | 159.7659 | 0.6426 |
| 6 minutes walk test (Postsurgery 8 weeks) | 0 | 0 | 771.4839 | 0.4372 |
| Anxiety (Presurgery) | 0.18 | 1 | 0.8323 | 0.6754 |
| Anxiety (Postsurgery 4 weeks) | - | - | - | - |
| Anxiety (Postsurgery 8 weeks) | 0.00 | 0 | 0 | 0.7056 |
| Depressive (Presurgery) | 0.40 | 1 | 0.3933 | 0.5278 |
| Depressive (Postsurgery 4 weeks) | - | - | - | - |
| Depressive (Postsurgery 8 weeks) | 0.00 | 0 | 0.0139 | 0.6263 |

**Table 5.3.1 Details of SIDE splitting results (Postoperative complications)**

| **Comparison** | **k** | **prop** | **nma** | **direct** | **indir.** | **RoR** | **z** | **p-value** |
| --- | --- | --- | --- | --- | --- | --- | --- | --- |
| BT vs CON | 1 | 1 | 0.257 | 0.257 | NA | NA | NA | NA |
| BT vs EX | 0 | 0 | 0.5339 | NA | 0.5339 | NA | NA | NA |
| BT vs Mul | 0 | 0 | 0.5507 | NA | 0.5507 | NA | NA | NA |
| BT vs NU | 0 | 0 | 0.3417 | NA | 0.3417 | NA | NA | NA |
| EX vs CON | 5 | 0.8 | 0.4814 | 0.4509 | 0.6229 | 0.7239 | -0.31 | 0.7573 |
| Mul vs CON | 10 | 0.9 | 0.4667 | 0.4813 | 0.3484 | 1.3814 | 0.31 | 0.7573 |
| NU vs CON | 3 | 1 | 0.7522 | 0.7522 | NA | NA | NA | NA |
| EX vs Mul | 1 | 0.3 | 1.0314 | 1.2941 | 0.9368 | 1.3814 | 0.31 | 0.7573 |
| EX vs NU | 0 | 0 | 0.64 | NA | 0.64 | NA | NA | NA |
| Mul vs NU | 0 | 0 | 0.6205 | NA | 0.6205 | NA | NA | NA |

**Table 5.3.2 Details of SIDE splitting results (Hospital day)**

| **Comparison** | **k** | **prop** | **nma** | **direct** | **indir.** | **Diff** | **z** | **p-value** |
| --- | --- | --- | --- | --- | --- | --- | --- | --- |
| BT vs CON | 1 | 0.96 | -1.1944 | -1 | -6.1424 | 5.1424 | 0.93 | 0.3509 |
| BT vs EX | 1 | 0.05 | -0.4388 | -5.3 | -0.1576 | -5.1424 | -0.93 | 0.3509 |
| BT vs Mul | 0 | 0 | -0.0232 | NA | -0.0232 | NA | NA | NA |
| BT vs NU | 0 | 0 | -0.5187 | NA | -0.5187 | NA | NA | NA |
| EX vs CON | 4 | 0.98 | -0.7556 | -0.8424 | 4.3 | -5.1424 | -0.93 | 0.3509 |
| Mul vs CON | 13 | 1 | -1.1712 | -1.1712 | NA | NA | NA | NA |
| NU vs CON | 3 | 1 | -0.6756 | -0.6756 | NA | NA | NA | NA |
| EX vs Mul | 0 | 0 | 0.4156 | NA | 0.4156 | NA | NA | NA |
| EX vs NU | 0 | 0 | -0.0799 | NA | -0.0799 | NA | NA | NA |
| Mul vs NU | 0 | 0 | -0.4955 | NA | -0.4955 | NA | NA | NA |

**Table 5.3.3 Details of SIDE splitting results (6 minutes walk test-Presurgery)**

| **comparison** | **k** | **prop** | **nma** | **direct** | **indir.** | **Diff** | **z** | **p-value** |
| --- | --- | --- | --- | --- | --- | --- | --- | --- |
| EX vs CON | 5 | 0.49 | 11.1453 | 15.6403 | 6.824 | 8.8164 | 0.38 | 0.7065 |
| Mul vs CON | 8 | 0.89 | 27.218 | 26.224 | 35.0403 | -8.8164 | -0.38 | 0.7065 |
| NU vs CON | 1 | 1 | 19.6 | 19.6 | NA | NA | NA | NA |
| EX vs Mul | 1 | 0.62 | -16.0726 | -19.4 | -10.5836 | -8.8164 | -0.38 | 0.7065 |
| EX vs NU | 0 | 0 | -8.4547 | NA | -8.4547 | NA | NA | NA |
| Mul vs NU | 0 | 0 | 7.618 | NA | 7.618 | NA | NA | NA |

**Table 5.3.4 Details of SIDE splitting results (6 minutes walk test (postsurgery 4 weeks))**

| **Comparison** | **k** | **prop** | **nma** | **direct** | **indir.** | **Diff** | **z** | **p-value** |
| --- | --- | --- | --- | --- | --- | --- | --- | --- |
| EX vs CON | 1 | 1 | 65 | 65 | NA | NA | NA | NA |
| Mul vs CON | 8 | 1 | 19.2237 | 19.2237 | NA | NA | NA | NA |
| NU vs CON | 1 | 1 | 17.6 | 17.6 | NA | NA | NA | NA |
| EX vs Mul | 0 | 0 | 45.7763 | NA | 45.7763 | NA | NA | NA |
| EX vs NU | 0 | 0 | 47.4 | NA | 47.4 | NA | NA | NA |
| Mul vs NU | 0 | 0 | 1.6237 | NA | 1.6237 | NA | NA | NA |

**Table 5.3.5 Details of SIDE splitting results (6 minutes walk test (Postsurgery 8 weeks))**

| **Comparison** | **k** | **prop** | **nma** | **direct** | **indir.** | **Diff** | **z** | **p-value** |
| --- | --- | --- | --- | --- | --- | --- | --- | --- |
| EX vs CON | 0 | 0 | 2.107 | NA | 2.107 | NA | NA | NA |
| Mul vs CON | 5 | 1 | 24.407 | 24.407 | NA | NA | NA | NA |
| EX vs Mul | 1 | 1 | -22.3 | -22.3 | NA | NA | NA | NA |

**Table 5.3.6 Details of SIDE splitting results (Anxiety (Presurgery))**

| **Comparison** | **k** | **prop** | **nma** | **direct** | **indir.** | **Diff** | **z** | **p-value** |
| --- | --- | --- | --- | --- | --- | --- | --- | --- |
| EX vs CON | 1 | 0.54 | -0.2598 | 0 | -0.5653 | 0.5653 | 0.47 | 0.6413 |
| Mul vs CON | 6 | 0.92 | -0.6913 | -0.7371 | -0.1718 | -0.5653 | -0.47 | 0.6413 |
| EX vs Mul | 1 | 0.54 | 0.4315 | 0.1718 | 0.7371 | -0.5653 | -0.47 | 0.6413 |

**Table 5.3.7 Details of SIDE splitting results (Anxiety (Postsurgery 8 weeks))**

| **Comparison** | **k** | **prop** | **nma** | **direct** | **indir.** | **Diff** | **z** | **p-value** |
| --- | --- | --- | --- | --- | --- | --- | --- | --- |
| EX vs CON | 1 | 1 | -0.5351 | -0.5351 | NA | NA | NA | NA |
| Mul vs CON | 3 | 1 | -0.0827 | -0.0827 | NA | NA | NA | NA |
| EX vs Mul | 0 | 0 | -0.4524 | NA | -0.4524 | NA | NA | NA |

**Table 5.3.8 Details of SIDE splitting results (Depressive (Presurgery))**

| **Comparison** | **k** | **prop** | **nma** | **direct** | **indir.** | **Diff** | **z** | **p-value** |
| --- | --- | --- | --- | --- | --- | --- | --- | --- |
| EX vs CON | 2 | 0.68 | -0.2271 | -0.0559 | -0.5959 | 0.54 | 0.68 | 0.4973 |
| Mul vs CON | 6 | 0.9 | -0.3661 | -0.4223 | 0.1177 | -0.54 | -0.68 | 0.4973 |
| EX vs Mul | 1 | 0.42 | 0.139 | -0.1736 | 0.3664 | -0.54 | -0.68 | 0.4973 |

**Table 5.3.9 Details of SIDE splitting results (Depressive (Postsurgery 8 weeks))**

| **Comparison** | **k** | **prop** | **nma** | **direct** | **indir.** | **Diff** | **z** | **p-value** |
| --- | --- | --- | --- | --- | --- | --- | --- | --- |
| EX vs CON | k | prop | nma | direct | indir. | Diff | z | p-value |
| Mul vs CON | 0 | 0 | -0.0871 | NA | -0.0871 | NA | NA | NA |
| EX vs Mul | 3 | 1 | 0.0232 | 0.0232 | NA | NA | NA | NA |

NA not available, k Number of studies providing direct evidence, prop Direct evidence proportion, nma Estimated treatment effect in network meta-analysis, direct Estimated treatment effect derived from direct evidence, indir. Estimated treatment effect derived from indirect evidence, ROR Ratio of Ratios (direct versus indirect), Diff direct versus indirect, z z-value of test for disagreement (direct versus indirect), p p-value of test for disagreement (direct versus indirect).

# Supplementary 6: Network plot

Figure 6.1: Network plot of postoperative complications. The size of the nodes corresponds to the number of participants randomized to each prehabilitation type. Prehabilitation type with direct comparisons are linked with a line; its thickness corresponds to the number of trials evaluating the comparison. BT breath training, CON standard care, EX exercise, Mul multimodal prehabilitation programme (exercise, nutritional, and anxiety-reduction strategies), NU nutritional intervention.

Figure 6.2: Network plot of hospital day. The size of the nodes corresponds to the number of participants randomized to each prehabilitation programme type. prehabilitation programme type with direct comparisons are linked with a line; its thickness corresponds to the number of trials evaluating the comparison. BT breath training, CON standard care, EX exercise, Mul multimodal prehabilitation programme (exercise, nutritional, and anxiety-reduction strategies), NU nutritional intervention.

Figure 6.3: Network plot of 6 minutes walk test (Presurgery). The size of the nodes corresponds to the number of participants randomized to each prehabilitation type. Prehabilitation type with direct comparisons are linked with a line; its thickness corresponds to the number of trials evaluating the comparison.

Figure 6.4: Network plot of 6 minutes walk test (Postsurgery 4 weeks). The size of the nodes corresponds to the number of participants randomized to each prehabilitation type. Prehabilitation type with direct comparisons are linked with a line; its thickness corresponds to the number of trials evaluating the comparison.

Figure 6.5: Network plot of 6 minutes walk test (Postsurgery 8 weeks). The size of the nodes corresponds to the number of participants randomized to each prehabilitation type. Prehabilitation type with direct comparisons are linked with a line; its thickness corresponds to the number of trials evaluating the comparison.

Figure 6.6: Network plot of Anxiety (Presurgery). The size of the nodes corresponds to the number of participants randomized to each prehabilitation type. Prehabilitation type with direct comparisons are linked with a line; its thickness corresponds to the number of trials evaluating the comparison.

Figure 6.7: Network plot of Anxiety (Postsurgery 8 weeks). The size of the nodes corresponds to the number of participants randomized to each prehabilitation type. Prehabilitation type with direct comparisons are linked with a line; its thickness corresponds to the number of trials evaluating the comparison.

Figure 6.8: Network plot of Depressive (Presurgery). The size of the nodes corresponds to the number of participants randomized to each prehabilitation type. Prehabilitation type with direct comparisons are linked with a line; its thickness corresponds to the number of trials evaluating the comparison.

Figure 6.9: Network plot of Depressive (Postsurgery 8 weeks). The size of the nodes corresponds to the number of participants randomized to each prehabilitation type. Prehabilitation type with direct comparisons are linked with a line; its thickness corresponds to the number of trials evaluating the comparison

# Supplementary 7: League table

Table 7.1 The league table of postoperative complications

| **BT**  **(0.87)** | NA | NA | NA | 0.26 (0.04; 1.57) |
| --- | --- | --- | --- | --- |
| 0.55 (0.08; 3.71) | **Mul**  **(0.64)** | 0.77 (0.14; 4.30) | NA | **0.48 (0.26; 0.91)** |
| 0.53 (0.07; 3.91) | 0.97 (0.38; 2.47) | **EX**  **(0.61)** | NA | 0.45 (0.18; 1.13) |
| 0.34 (0.04; 2.88) | 0.62 (0.17; 2.22) | 0.64 (0.16; 2.58) | **NU**  **(0.34)** | 0.75 (0.24; 2.32) |
| 0.26 (0.04; 1.57) | **0.47 (0.26; 0.85)** | 0.48 (0.21; 1.10) | 0.75 (0.24; 2.32) | **CON**  **(0.11)** |

All results are presented in the form of OR (95% CrI). Treatment types are ranked according to the SUCRA for postoperative complications with the best from left to right. The results of the network meta-analysis are showed in the lower left part, and results from pairwise comparisons in the upper right half (if available). Cells shown in bold indicate significant results. NA not available, OR odds ratio, CrI Credible Interval, BT Breathing training, Mul Multimodal program (involving exercise, nutritional, and psychological), NU Nutrition intervention, CON control group (standard care).

Table 7.2 The league table of hospital day

| **Mul**  **(0.72)** | NA | NA | NA | **-1.17 ( -1.77; -0.57)** |
| --- | --- | --- | --- | --- |
| 0.02 (-2.12; 2.17) | **BT**  **(0.66)** | -5.30 (-15.81; 5.21) | NA | -1.00 ( -3.10; 1.10) |
| -0.42 (-1.93; 1.10) | -0.44 (-2.90; 2.02) | **EX**  **(0.51)** | NA | -0.84 ( -2.25; 0.56) |
| -0.50 (-2.61; 1.62) | -0.52 (-3.41; 2.37) | -0.08 (-2.54; 2.38) | **NU**  **(0.48)** | -0.68 ( -2.70; 1.35) |
| **-1.17 (-1.77; -0.57)** | -1.19 (-3.26; 0.87) | -0.76 (-2.15; 0.64) | -0.68 (-2.70; 1.35) | **CON**  **(0.13)** |

All results are presented in the form of MD (95% CrI). Treatment types are ranked according to the SUCRA for hospital day with the best from left to right. The results of the network meta-analysis are showed in the lower left part, and results from pairwise comparisons in the upper right half (if available). Cells shown in bold indicate significant results. NA not available, MD mean difference, CrI Credible Interval, BT Breathing training, Mul Multimodal program (involving exercise, nutritional, and psychological), NU Nutrition intervention, CON control group (standard care).

Table 7.3 The league table of 6 minutes walk test (Presurgery)

| **Mul**  **(0.85)** | NA | 19.40 ( -8.79; 47.59) | **26.22 ( 10.82; 41.63)** |
| --- | --- | --- | --- |
| 7.62 (-37.88; 53.12) | **NU**  **(0.61)** | NA | 19.60 (-23.52; 62.72) |
| 16.07 ( -6.17; 38.32) | 8.45 (-40.39; 57.30) | **EX**  **(0.43)** | 15.64 (-17.12; 48.40) |
| **27.22 ( 12.71; 41.73)** | 19.60 (-23.52; 62.72) | 11.15 (-11.79; 34.08) | **CON**  **(0.12)** |

All results are presented in the form of MD (95% CrI). Treatment types are ranked according to the SUCRA for 6 minutes walk test (presurgery) with the best from left to right. The results of the network meta-analysis are showed in the lower left part, and results from pairwise comparisons in the upper right half (if available). Cells shown in bold indicate significant results. NA not available, MD mean difference, CrI Credible Interval, BT Breathing training, Mul Multimodal program (involving exercise, nutritional, and psychological), NU Nutrition intervention, CON control group (standard care).

Table 7.4 The league table of 6 minutes walk test (Postsurgery 4 weeks)

| **EX**  **(0.74)** | NA | NA | 65.00 ( -85.88; 215.88) |
| --- | --- | --- | --- |
| 45.78 (-105.69; 197.24) | **Mul**  **(0.60)** | NA | **19.22 (5.94; 32.50)** |
| 47.40 (-120.70; 215.50) | 1.62 ( -73.68; 76.92) | **NU**  **(0.48)** | 17.60 ( -56.52; 91.72) |
| 65.00 ( -85.88; 215.88) | **19.22 (5.94; 32.50)** | 17.60 ( -56.52; 91.72) | **CON**  **(0.17)** |

All results are presented in the form of MD (95% CrI). Treatment types are ranked according to the SUCRA for 6 minutes walk test (postsurgery 4 weeks) with the best from left to right. The results of the network meta-analysis are showed in the lower left part, and results from pairwise comparisons in the upper right half (if available). Cells shown in bold indicate significant results. NA not available, MD mean difference, CrI Credible Interval, BT Breathing training, Mul Multimodal program (involving exercise, nutritional, and psychological), NU Nutrition intervention, CON control group (standard care).

Table 7.5 The league table of 6 minutes walk test (Postsurgery 8 weeks)

| **Mul**  **(0.86)** | 22.30 (-32.51; 77.11) | 24.41 ( -7.26; 56.08) |
| --- | --- | --- |
| 22.30 (-32.51; 77.11) | **EX**  **(0.37)** | NA |
| 24.41 ( -7.26; 56.08) | 2.11 (-61.19; 65.41) | **CON**  **(0.27)** |

All results are presented in the form of MD (95% CrI). Treatment types are ranked according to the SUCRA for 6 minutes walk test (postsurgery 8 weeks) with the best from left to right. The results of the network meta-analysis are showed in the lower left part, and results from pairwise comparisons in the upper right half (if available). Cells shown in bold indicate significant results. NA not available, MD mean difference, CrI Credible Interval, BT Breathing training, Mul Multimodal program (involving exercise, nutritional, and psychological), NU Nutrition intervention, CON control group (standard care).

Table 7.6 The league table of Anxiety (Presurgery)

| **Mul**  **(0.87)** | -0.17 (-1.78; 1.44) | **-0.74 (-1.41; -0.06)** |
| --- | --- | --- |
| -0.43 (-1.62; 0.75) | **EX**  **(0.45)** | -0.00 (-1.61; 1.61) |
| **-0.69 (-1.34; -0.04)** | -0.26 (-1.44; 0.93) | **CON**  **(0.18)** |

All results are presented in the form of SMD (95% CrI). Treatment types are ranked according to the SUCRA for anxiety (presurgery) with the best from left to right. The results of the network meta-analysis are showed in the lower left part, and results from pairwise comparisons in the upper right half (if available). Cells shown in bold indicate significant results. NA not available, SMD standard mean difference, CrI Credible Interval, BT Breathing training, Mul Multimodal program (involving exercise, nutritional, and psychological), NU Nutrition intervention, CON control group (standard care).

Table 7.7 The league table of Anxiety (Postsurgery 8 weeks)

| **Mul**  **(0.70)** | -0.07 (-0.44; 0.30) | -0.08 (-0.32; 0.15) |
| --- | --- | --- |
| -0.07 (-0.44; 0.30) | **EX**  **(0.44)** | NA |
| -0.08 (-0.32; 0.15) | -0.01 (-0.45; 0.43) | **CON**  **(0.36)** |

All results are presented in the form of SMD (95% CrI). Treatment types are ranked according to the SUCRA for anxiety (postsurgery 8 weeks) with the best from left to right. The results of the network meta-analysis are showed in the lower left part, and results from pairwise comparisons in the upper right half (if available). Cells shown in bold indicate significant results. NA not available, SMD standard mean difference, CrI Credible Interval, BT Breathing training, Mul Multimodal program (involving exercise, nutritional, and psychological), NU Nutrition intervention, CON control group (standard care).

Table 7.8 The league table of depressive (Presurgery)

| **Mul**  **(0.79)** | 0.17 (-1.01; 1.36) | -0.42 (-0.93; 0.08) |
| --- | --- | --- |
| -0.14 (-0.91; 0.63) | **EX**  **(0.55)** | -0.06 (-0.93; 0.82) |
| -0.37 (-0.84; 0.11) | -0.23 (-0.95; 0.50) | **CON**  **(0.17)** |

All results are presented in the form of SMD (95% CrI). Treatment types are ranked according to the SUCRA for depressive (Presurgery) with the best from left to right. The results of the network meta-analysis are showed in the lower left part, and results from pairwise comparisons in the upper right half (if available). Cells shown in bold indicate significant results. NA not available, SMD standard mean difference, CrI Credible Interval, BT Breathing training, Mul Multimodal program (involving exercise, nutritional, and psychological), NU Nutrition intervention, CON control group (standard care).

Table 7.9 The league table of depressive (Postsurgery 8 weeks)

| **EX**  **(0.66)** | NA | -0.11 (-0.55; 0.33) |
| --- | --- | --- |
| -0.09 (-0.60; 0.43) | **CON**  **(0.47)** | -0.02 (-0.29; 0.25) |
| -0.11 (-0.55; 0.33) | -0.02 (-0.29; 0.25) | **Mul**  **(0.37)** |

All results are presented in the form of SMD (95% CrI). Treatment types are ranked according to the SUCRA for depressive (Postsurgery 8 weeks) with the best from left to right. The results of the network meta-analysis are showed in the lower left part, and results from pairwise comparisons in the upper right half (if available). Cells shown in bold indicate significant results. NA not available, SMD standard mean difference, CrI Credible Interval, BT Breathing training, Mul Multimodal program (involving exercise, nutritional, and psychological), NU Nutrition intervention, CON control group (standard care).

# Supplementary 8: Publication bias

Figure 8.1 The funnel plot of postoperative complications. The result of Egger test showed the p=454. Therefore, no small study effect was found for the postoperative complications.

Figure 8.2 The funnel plot of hospital day. The result of Egger test showed the p=0.150. Therefore, no small study effect was found for the hospital day.

Figure 8.3 The funnel plot of 6 minutes walk test (Presurgery). The result of Egger test showed the p=0.548. Therefore, no small study effect was found for the 6 minutes walk test (Presurgery)

Figure 8.4 The funnel plot of 6 minutes walk test (Postsurgery 4 weeks). The result of Egger test showed the p=0.106. Therefore, no small study effect was found for 6 minutes walk test (Postsurgery 4 weeks)

Figure 8.5 The funnel plot of 6 minutes walk test (Postsurgery 8 weeks). The result of Egger test showed the p=0.851. Therefore, no small study effect was found for 6 minutes walk test (Postsurgery 8 weeks)

Figure 8.6 The funnel plot of anxiety (Presurgery). The result of Egger test showed the p=0.111. Therefore, no small study effect was found for the anxiety (Presurgery)

Figure 8.7 The funnel plot of anxiety (Prostsurgery 4 weeks). The result of Egger test showed the p=0.842. Therefore, no small study effect was found for the anxiety (Prostsurgery 4 weeks).

Figure 8.8 The funnel plot of anxiety (Prostsurgery 8 weeks). The result of Egger test showed the p=0.911. Therefore, no small study effect was found for the anxiety (Prostsurgery 8 weeks).

Figure 8.9 The funnel plot of depressive (Presurgery). The result of Egger test showed the p=0.272. Therefore, no small study effect was found for the depressive (Presurgery).

Figure 8.10 The funnel plot of depressive (Postsurgery 4 weeks). The result of Egger test showed the p=0.472. Therefore, no small study effect was found for the depressive (Postsurgery 4 weeks).

Figure 8.11 The funnel plot of depressive (Postsurgery 8 weeks). The result of Egger test showed the p=0.056. Therefore, no small study effect was found for the depressive (Postsurgery 8 weeks)

Supplementary 9: Forest plot of meta-analysis for pairwise comparisons

Figure 9.1 Forest plot of the effect of Mul on patients' anxiety symptoms 4 weeks after surgery. SMD standard mean difference, CI Credible Interval.

Figure 9.2 Forest plot of the effect of Mul on patients' depressive symptoms 4 weeks after surgery. SMD standard mean difference, CI Credible Interval.

Supplementary 10: Sensitivity analysis

After we excluded two high-risk studies, the results for the primary outcome remained consistent with the original results (Figure 10.1-2).

Figure 10.1: Forest plot of studies at moderate or low RoB (postoperative complications). OR odds ratio, CrI Credible Interval, BT Breathing training, Mul Multimodal program (involving exercise, nutritional, and psychological), NU Nutrition intervention, CON control group (standard care).

Figure 10.1: Forest plot of studies at moderate or low RoB (the length of hospital stay). MD mean differenct, CrI Credible Interval, BT Breathing training, Mul Multimodal program (involving exercise, nutritional, and psychological), NU Nutrition intervention, CON control group (standard care).
